# Trodusquemine displaces protein misfolded oligomers from cell membranes and abrogates their cytotoxicity through a generic mechanism

**DOI:** 10.1038/s42003-020-01140-8

**Published:** 2020-08-13

**Authors:** Ryan Limbocker, Benedetta Mannini, Francesco S. Ruggeri, Roberta Cascella, Catherine K. Xu, Michele Perni, Sean Chia, Serene W. Chen, Johnny Habchi, Alessandra Bigi, Ryan P. Kreiser, Aidan K. Wright, J. Alex Albright, Tadas Kartanas, Janet R. Kumita, Nunilo Cremades, Michael Zasloff, Cristina Cecchi, Tuomas P. J. Knowles, Fabrizio Chiti, Michele Vendruscolo, Christopher M. Dobson

**Affiliations:** 1 Centre for Misfolding Diseases, Department of Chemistry, University of Cambridge, Cambridge, CB2 1EW UK; 2 Department of Chemistry and Life Science, United States Military Academy, West Point, NY 10996 USA; 3 Department of Experimental and Clinical Biomedical Science, University of Florence, 50134 Florence, Italy; 4 Institute for Biocomputation and Physics of Complex Systems (BIFI)-Joint Unit BIFI-IQFR (CSIC), University of Zaragoza, 50018 Zaragoza, Spain; 5 MedStar-Georgetown Transplant Institute, Georgetown University School of Medicine, Washington, DC 20010 USA; 6 Cavendish Laboratory, Department of Physics, University of Cambridge, Cambridge, CB3 0HE UK

**Keywords:** Biophysics, Chemical biology

## Abstract

The onset and progression of numerous protein misfolding diseases are associated with the presence of oligomers formed during the aberrant aggregation of several different proteins, including amyloid-β (Aβ) in Alzheimer’s disease and α-synuclein (αS) in Parkinson’s disease. These small, soluble aggregates are currently major targets for drug discovery. In this study, we show that trodusquemine, a naturally-occurring aminosterol, markedly reduces the cytotoxicity of αS, Aβ and HypF-N oligomers to human neuroblastoma cells by displacing the oligomers from cell membranes in the absence of any substantial morphological and structural changes to the oligomers. These results indicate that the reduced toxicity results from a mechanism that is common to oligomers from different proteins, shed light on the origin of the toxicity of the most deleterious species associated with protein aggregation and suggest that aminosterols have the therapeutically-relevant potential to protect cells from the oligomer-induced cytotoxicity associated with numerous protein misfolding diseases.

## Abstract (web summary)

Limbocker et al. show that trodusquemine, an aminosterol, reduces the cytotoxicity of protein misfolded oligomers by displacing them from cell membranes in the absence of any overt structural/ morphological changes in them. This mechanism appears to be general, as they test it for oligomers of αS, Aβ and the model protein HypF-N to human neuroblastoma cells.

## Introduction

The deposition of a range of peptides and proteins as pathological aggregates plays a central role in the etiology of over fifty human disorders, including the amyloid-β protein (Aβ) in Alzheimer’s disease and α-synuclein (αS) in Parkinson’s disease^1–3^. During the aggregation reaction, monomeric proteins that are normally soluble misfold, self-associate and ultimately form amyloid fibrils with a characteristic cross-β structure, through a mechanism that has been shown to be common to a wide range of proteins^1–3^. Increasing evidence suggests that the small soluble oligomers formed during the aggregation process of such amyloidogenic proteins are highly likely to play an important role in the onset and progression of diseases^3–6^. Oligomers are therefore major targets for drug discovery against protein misfolding diseases. Different mechanisms through which the oligomers exert their toxicity have been reported, for example by impairing and sequestering functional forms of proteins^7^, compromising the integrity of lipid membranes^8,9^, interacting with membrane proteins, particularly calcium channels^10^, and stimulating the immune response^11^.

The application of a highly sensitive kinetic assay to characterize the aggregation behavior of the 42-residue form of the amyloid-β protein (Aβ_42_)^12–14^ has suggested the possibility of reducing the toxicity inherent to protein aggregation by targeting the generation of oligomers^15^. This methodology has enabled the detailed evaluation of compounds, including molecular chaperones, antibodies, and small molecules, for their abilities to inhibit the aggregation process of Aβ_42_ in a specific manner so that the overall concentration of oligomers produced during the process is reduced. Such compounds have the potential to serve in a therapeutic capacity by targeting specifically the microscopic steps of Aβ_42_ that are responsible for oligomer production and proliferation^15–19^. Similar strategies have been employed to identify species that inhibit oligomer formation by αS, in particular by targeting the microscopic step of fibril amplification^20–22^.

We have previously reported that the small molecule trodusquemine, an aminosterol first discovered in dogfish sharks and initially investigated for its therapeutic potential against bacterial infection, obesity, cancer, and anxiety disorders^23–26^, and as a stimulant of tissue regeneration^27^, acts as a potent inhibitor of αS aggregation by reducing the rates associated with the lipid-induced nucleation and fibril amplification steps in the aggregation process^20^. In a surprising contrast, we also found that trodusquemine enhances the aggregation of Aβ_42_ by increasing the rate of monomer-dependent secondary nucleation and, to a lower extent, fibril elongation, while maintaining the rate of primary nucleation almost unaffected^28^. Despite the distinct mechanisms of the aggregation of αS and Aβ_42_ and the differing effects of trodusquemine on the underlying processes, however, these effects resulted in a lower steady-state concentration of oligomers in both cases, in spite of the accelerated overall aggregation process in the case of Aβ_42_^28^. Moreover, this molecule was found to suppress the toxic effects of both αS and Aβ_42_ oligomers in cultured human neuroblastoma cells by markedly reducing the affinity of these aggregates for the cell membrane^20,28^. We also found that squalamine, an aminosterol comparable in structure to trodusquemine but with one less positive charge in its polyamine chain, similarly reduces the toxicity of αS oligomers to cells by their displacement from cell membranes^22^. Collectively, the studies on trodusquemine and squalamine raise the hypothesis that the aminosterol family of molecules can protect the cells from the deleterious interaction of misfolded oligomeric species^20,22,28^. Furthermore, trodusquemine exhibits an apparently enhanced potency over squalamine against the aberrant behavior of αS, as manifested by its ability also to inhibit more strongly the fibril amplification process, and to reduce the toxicity toward human neuroblastoma cells at lower concentrations. In addition, both aminosterols lower the toxicity caused by αS aggregation in a worm model of Parkinson’s disease^20,22^, and trodusquemine lessened the toxicity related to Aβ_42_ aggregation in a worm model of Alzheimer’s disease^28^.

In the present work, we studied the general mechanism of action of trodusquemine at the molecular level on a range of oligomeric systems. In particular, we investigated oligomers of αS and Aβ due to their roles in Parkinson’s and Alzheimer’s diseases, respectively. We used oligomers of Aβ_40_ in a recently characterized form stabilized by zinc ions^29^, which are distinct from the Aβ_42_ ADDLs described previously^28^. In addition, we also studied oligomeric species produced from the N-terminal domain of the HypF protein from *E. coli* (HypF-N)^8,30^ in order to explore the effects of trodusquemine on another well-characterized model of misfolded protein oligomers. We also explored the effects of trodusquemine on the morphological and structural properties of the oligomers thought to be responsible for mediating their toxicity. Our results reveal that trodusquemine protects cultured neuroblastoma cells from the toxicity induced by all the types of oligomers that we studied through markedly attenuating their interaction with cell membranes in the absence of any detectable structural changes and supramolecular reorganization. On the basis of these findings, we suggest that aminosterols and other molecules that act in a similar manner may function through a generic mechanism as therapeutic agents to combat a variety of human protein misfolding disorders.

## Results

### Trodusquemine inhibits oligomer toxicity toward cells

Because of the heterogeneous and transient nature of protein misfolded oligomers, it is typically necessary to isolate and stabilize them through physical or chemical means to study their physicochemical, structural and biological properties. For example, only a maximum of 1% of the total monomer concentration for Aβ is oligomeric at the half-point of the in vitro aggregation reaction when carried out at the concentrations used in this work^31,32^. A key advantage of the methodologies employed herein for the production of αS, Aβ_40_, or HypF-N oligomers is that they are highly reproducible and yield markedly more homogeneous and stable populations in comparison to the dynamical, metastable, and heterogeneous aggregates that are formed during an endogenous aggregation reaction^8,29,33^.

**Fig. 1:**
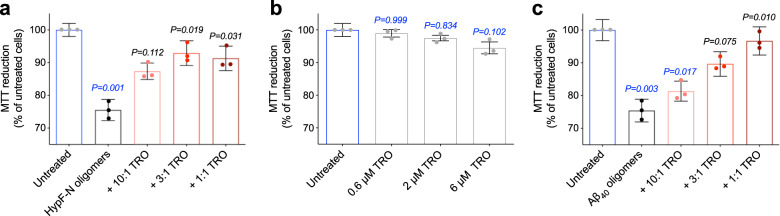
Trodusquemine reduces the toxicity of HypF-N and Aβ_40_ oligomers toward human neuroblastoma cells. **a** Oligomers of HypF-N (6 µM, monomer equivalents) were resuspended in the cell culture medium in the absence (black) and presence of 10:1, 3:1, and 1:1 (light to dark red) molar ratios of oligomers to trodusquemine (TRO), incubated for 1 h at 37 °C, and subsequently added to the cell culture medium of SH-SY5Y cells for 24 h. Untreated cells exposed only to cell culture medium are shown for reference (blue). **b** Cells were also treated with the corresponding concentrations of trodusquemine (0.6, 2, and 6 µM; gray bars) pre-incubated in the absence of oligomers under the same conditions. **c** Aβ_40_ oligomers stabilized by Zn^2+^ (5 µM, monomer equivalents) were incubated and administered to cells under the conditions described in **a**. Comparisons were carried out by one-way ANOVA relative to untreated cells (indicated by the *P* values in blue text) and cells treated with oligomers alone (indicated by the *P* values in black text). All bars indicate mean ± standard error of the mean (s.e.m.) of *n* = 3 biologically independent experiments (dots).

We began our cellular investigations by determining the ability of toxic type A HypF-N oligomers^8^ (6 µM, monomer equivalents), typically having a height of 2–6 nm, as determined with atomic force microscopy (AFM)^8^, to reduce the viability of SH-SY5Y cells. The solutions containing the oligomers and their respective controls were incubated for 1 h at 37 °C in the absence and presence of 10:1, 3:1, and 1:1 ratios of oligomers to trodusquemine, and were subsequently added to the culture medium of the cells for 24 h (Fig. 1a). In the presence of the HypF-N oligomers alone, the ability of cells to reduce MTT was decreased to 76 ± 3% of that of untreated cells, indicating a significant decrease in viability (*P* = 0.001, one-way ANOVA relative to untreated cells), in agreement with previous studies^8^. The addition of trodusquemine, however, was observed to improve significantly the viability of the cells, such that the toxicity of the oligomers was nearly eliminated when incubated with an equimolar concentration of the small molecule (*P* = 0.031, one-way ANOVA relative to cells treated with oligomers; Fig. 1a). Indeed, the small decrease in cellular viability at the highest concentration of trodusquemine used in this study (6 µM) to 91 ± 4% of that of untreated cells was essentially identical to that observed in cells treated with corresponding concentrations of trodusquemine in the absence of oligomers, to 94 ± 2% of that of untreated cells (Fig. 1b). Cells were also incubated under the same conditions with 2% triton X-100 as a positive control in the MTT assay, and cell viability was quantified to be 8.0 ± 0.3% of untreated cells after being lysed (Supplementary Fig. 1).

In a second set of experiments, we investigated the effects of trodusquemine on zinc-stabilized Aβ_40_ oligomers^29^. Aβ_40_ oligomers stabilized by zinc ions represent a valuable oligomeric model for studying the acute toxicity associated with Alzheimer’s disease because they are not stabilized by an invasive chemical-physical modification, but rather by a metabolite that is naturally present at synapses. This model may therefore resemble populations of oligomers endogenously occurring in the brain^29^. While Aβ_40_ is more abundant than Aβ_42_ (80–90% versus 5–10% of total Aβ in the brain^1,3,34,35^), the more hydrophobic Aβ_42_ has been shown to be more toxic and aggregation prone^35,36^, and we have previously studied in detail the effects of trodusquemine on Aβ_42_^28^.

In these measurements, zinc-stabilized Aβ_40_ oligomers^29^ (5 µM, monomer equivalents), typically having a height of 1–5 nm as determined with AFM^29^, were pre-incubated in a similar manner to those of HypF-N, again for 1 h at 37 °C in the absence and presence of trodusquemine at ratios of the oligomers to trodusquemine of 10:1, 3:1, and 1:1, and then added to the culture medium of the cells for 24 h (Fig. 1c). In the presence of Aβ_40_ oligomers, the ability of cells to reduce MTT was decreased to 75 ± 3% of untreated cells, indicating a significant decrease in viability (*P* = 0.003, one-way ANOVA relative to untreated cells). As was observed in the case of HypF-N oligomers, the addition of increasing concentrations of trodusquemine markedly improved the viability of cells in a dose-dependent manner, and cells treated with oligomers and an equimolar concentration of the molecule had a viability of 97 ± 4% of untreated cells (*P* = 0.010, one-way ANOVA relative to cells treated with oligomers) (Fig. 1c), a value that is essentially identical to that of cells treated with 6 µM trodusquemine alone (Fig. 1b).

Previously, we reported that the viability of SH-SY5Y cells measured by the MTT test in the presence of toxic, 4.3 ± 0.9 nm in height as measured with AFM^33^, type B αS oligomers (0.3 µM, monomer equivalents) was reduced to 79 ± 2% in the absence of trodusquemine, and increased with a well-defined dose dependence to 99 ± 2% upon the addition of an equimolar concentration of trodusquemine^20^. Of note, it has previously been demonstrated that these concentrations of trodusquemine do not displace monomeric αS from membranes^20^, suggesting that physiologically relevant concentrations of the molecule would not impact the normal association of monomeric αS with cell membranes. Collectively, therefore, cell viability was recovered to 91 ± 4%, 97 ± 4%, and 99 ± 2% of untreated cells in the presence of a 1:1 ratio of trodusquemine to oligomers for HypF-N, Aβ_40_ and αS oligomers, respectively, suggesting that the degree of protection of the cells is independent of the oligomer type.

The oligomer concentrations administered to cells of 6 µM, 5 µM, and 0.3 µM for HypF-N, zinc-stabilized Aβ_40_, and αS oligomers, respectively, were selected based on preliminary observations that oligomer toxicity peaks at these concentrations. To illustrate this point, we incubated human neuroblastoma cells with 0, 1, 6, 12, 24, and 48 µM concentrations of HypF-N oligomers, 0, 0.5, 1, 2.5, 5, 10, 20, 30, and 40 µM concentrations of zinc-stabilized Aβ_40_ oligomers, and 0, 0.03, 0.3, and 3 µM concentrations of αS oligomers (Supplementary Fig. 2). For these three systems, we found that the toxicity correlated non-linearly with the oligomer concentration, given that at high concentrations oligomers coalescence into larger, less toxic aggregates. Also in consideration of the observation that trodusquemine is soluble and monomeric at a concentration of 10 µM in phosphate-buffered saline solution^20^, we elected to use the aforementioned concentrations of oligomers in cells and in vitro.

### Oligomer displacement from membranes by a generic mechanism

The affinity of misfolded protein oligomers for cell membranes has been shown to correlate closely with the extent of their ability to induce cellular toxicity^37,38^. In the light of this connection, and of previous findings indicating that trodusquemine can displace αS and Aβ_42_ oligomers from membranes^20,28^, we performed experiments using confocal microscopy to probe the interactions between the stable oligomers of HypF-N and Aβ_40_ with the membranes of SH-SY5Y neuroblastoma cells to determine if the mechanism of action of trodusquemine is generic to multiple types of oligomers. The cells were treated for 15 min at 37 °C with HypF-N oligomers (6 µM, monomer equivalents) or Aβ_40_ oligomers (5 µM, monomer equivalents) in the absence and presence of 10:1, 3:1, and 1:1 ratios of oligomers to trodusquemine. The binding of oligomers to the membranes was monitored using anti-HypF-N or 6E10 sequence specific anti-Aβ antibodies (green channel) and wheat germ agglutinin (red channel) to label the oligomers and membranes, respectively, as previously described^22^. Cells were monitored at apical planes to avoid visualizing oligomers that may have internalized into the cytosol.

**Fig. 2:**
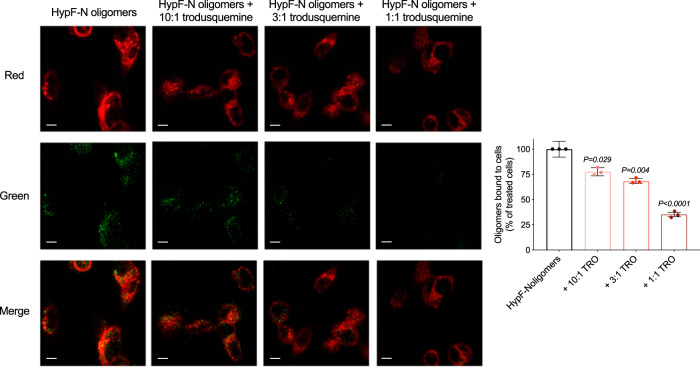
Trodusquemine reduces the membrane binding affinity of HypF-N oligomers to cultured human neuroblastoma cells. Representative confocal scanning microscopy images of the apical sections of SH-SY5Y cells treated for 15 min at 37 °C with HypF-N oligomers (6 µM) in the absence (black) or presence of 10:1, 3:1, and 1:1 ratios of HypF-N to trodusquemine (light to dark red). Red and green fluorescence indicates the cell membranes and the oligomers, respectively. The percentage of colocalization between membranes and HypF-N oligomers are shown. In all, 50–60 cells were analyzed per condition. Bars indicate the mean ± s.e.m. of *n* = 3 biologically independent experiments (dots). Statistical analyses were carried out by one-way ANOVA relative to cells treated with oligomers in the absence of trodusquemine. Scale bars, 10 µm.

**Fig. 3:**
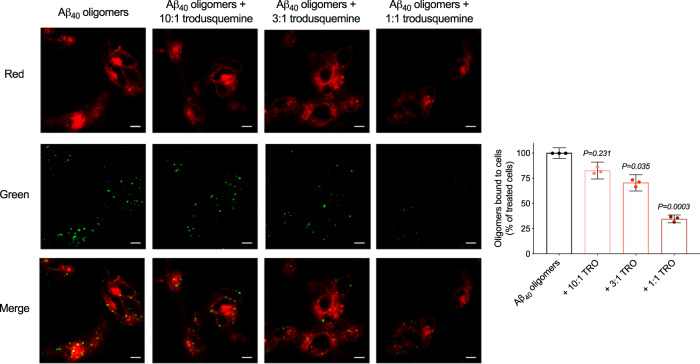
Trodusquemine reduces the membrane binding affinity of Aβ_40_ oligomers to cultured human neuroblastoma cells. Representative confocal scanning microscopy images of the apical sections of SH-SY5Y cells treated for 15 min at 37 °C with Aβ_40_ oligomers (5 µM) in the absence (black) and presence of 10:1, 3:1, and 1:1 ratios of Aβ_40_ to trodusquemine (light to dark red). Red and green fluorescence indicates the cell membranes and the oligomers, respectively. The percentage of colocalization between membranes and Aβ_40_ oligomers are shown. In all, 50–60 cells were analyzed per condition. Bars indicate the mean ± s.e.m. of *n* = 3 biologically independent experiments (dots). Statistical analyses were carried out by one-way ANOVA relative to cells treated with oligomers in the absence of trodusquemine. Scale bars, 10 µm.

Extensive oligomer binding to the apical planes of the cells was observed in the absence of trodusquemine for both HypF-N and Aβ_40_ oligomers, while in the presence of increasing concentrations of trodusquemine, the binding was decreased with a well-defined dose dependence (Figs. 2 and 3), as shown from measurements of the degree of colocalization between oligomers and membrane surfaces (Figs. 2 and 3). Indeed, at equimolar concentrations of trodusquemine and oligomers, oligomer binding was reduced by 65 ± 2% for HypF-N, 65 ± 4% for Aβ_40_ and 71 ± 2% for αS, the latter data being taken from a previous publication^20^. As observed for the reduction in oligomer toxicity, the degree of displacement is very similar across the proteins investigated here, suggesting that the protection of the cell membranes by trodusquemine is dominated by the displacement of the cytotoxic oligomers from the surface of the membrane.

Recent studies have demonstrated that the uptake of intrinsically disorder proteins, including Aβ, is a key step in its mechanism of toxicity^39–41^. It is therefore possible that the trodusquemine-induced displacement of oligomers from cell membranes could also suppress protein uptake and its associated toxicity, as the binding of the oligomers to the membrane is a necessary step for their internalization. Indeed, we observed minimal oligomer internalization in the presence of trodusquemine, when the cells were analyzed at median planes to focus on their interior (Supplementary Figs. 3 and 4).

To investigate further the mechanism by which the cell membrane is protected from oligomeric aggregates by trodusquemine, we next formed small unilamellar vesicles comprised of 1,2-dioleoyl-sn-glycero-3-phosphoethanolamine (DOPE), dioleoyl-sn-glycero-3-phospho-L-serine (DOPS) and 1,2-dioleoyl-sn-glycero-3-phosphocholine (DOPC) at a ratio of 5:3:2, which represents a physiologically relevant model membrane system^42–44^. Increasing concentrations of trodusquemine (ranging from 0–20 µM) were added to the vesicles at a total phospholipid concentration of 100 µM, and their size was monitored using static and dynamic light scattering (Supplementary Fig. 5). Both light scattering measurements clearly indicate that vesicle size increases with trodusquemine concentration. Indeed, the diameter was increased from ~22 nm to over 100 nm in the presence of a 5 µM concentration of trodusquemine. Based on these results, we hypothesize that the affinity of the oligomers for membranes may decrease in part as a consequence of the increasing stiffness of the membranes as the concentration of trodusquemine is increased.

### Trodusquemine exerts minimal effects on oligomer structures

In order to understand if trodusquemine exerts a protective effect by mechanisms other than displacement from cell membranes, we investigated whether or not the molecule has the ability to affect the structural properties of the different types of oligomers examined in this study up to the 1:1 molar ratio at which a full suppression of oligomer toxicity is observed.

**Fig. 4:**
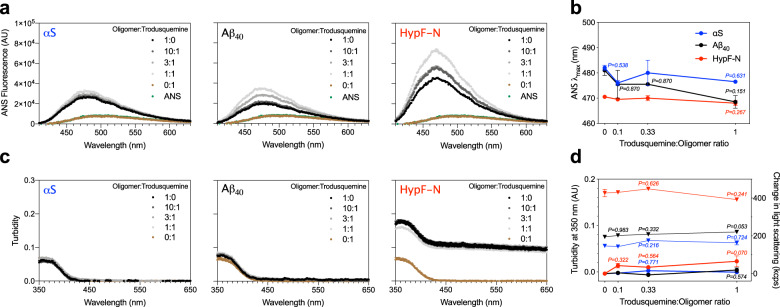
The concentrations of trodusquemine used in the cell experiments do not appreciably modify the biophysical properties of αS, Aβ_40_, and HypF-N oligomers. **a** Oligomers of αS, Aβ_40_ and HypF-N were incubated at a concentration of 5 µM in the absence (black) and presence of 10:1, 3:1, and 1:1 ratios of oligomers to trodusquemine (dark to light gray) and monitored for the binding of ANS. Free ANS (green) and ANS + 5 µM trodusquemine (brown) are shown for comparison. **b** The wavelength of maximum ANS fluorescence (*λ*_max_) for oligomers of αS (blue), Aβ_40_ (black), and HypF-N (red) in the presence of increasing concentrations of trodusquemine. **c** Turbidity absorbance measurements for the conditions described in **a**. **d** Turbidity values from **c** quantified at 350 nm (triangles, left y-axis). Change in static light scattering (kilocounts per second, kcps) after oligomer incubation in the presence of increasing concentrations of trodusquemine for the conditions described in **b** (dots, right *y*-axis). Static light scattering data indicate the mean ± s.e.m. of *n* = 3 technical replicates. In **a**–**c**, data indicate mean ± s.e.m. of *n* = 2 spectra. ANS and turbidity spectra are representative of three independent experiments that yielded consistent results. In **b**, **d**, *P* > 0.999 except where indicated (one-way ANOVA relative to oligomers in the absence of trodusquemine) with the aforementioned color codes and number of technical replicates.

A large body of literature has shown that the physicochemical properties of misfolded oligomers, in particular size and hydrophobicity, can be linked to their biological activity^3,8,45–47^. We therefore first sought to measure the solvent exposure of the oligomeric aggregates incubated in the presence of trodusquemine. 15 µM 8-anilinonaphthalene-1-sulfonate (ANS) was added to solutions containing the oligomers of αS, Aβ_40_, and HypF-N after their pre-incubation at a concentration of 5 µM in 20 mM Tris, 100 mM NaCl, pH 7.4, in the absence and presence of 10:1, 3:1, and 1:1 ratios of oligomers to trodusquemine (1 h at 20 °C). The binding of ANS to the oligomers of all three proteins in the absence of trodusquemine was observed to increase the fluorescence intensity of the dye and to shift the wavelength of maximum fluorescence to lower wavelengths (Fig. 4a), in agreement with previous observations^8,45,46^. Incubation of the oligomers with trodusquemine was found to increase slightly the maximum fluorescence intensity (Fig. 4a). The wavelength of maximum ANS fluorescence (*λ*_max_) was largely unchanged with increasing concentrations of trodusquemine (Fig. 4b). Taken together, the analyses indicate that trodusquemine is not appreciably altering the hydrophobicity of the oligomers at the concentrations used in the cell studies. As a control, the fluorescence intensity and *λ*_max_ value of free ANS remained unchanged at the highest concentration of trodusquemine (i.e. 5 µM) in the absence of oligomers (Fig. 4a).

We next sought to determine the impact of trodusquemine on the size of the oligomeric aggregates. The same samples as those used in the ANS binding experiments were examined, and incubation of the various oligomers with trodusquemine was not found to generate an observable increase in turbidity, as shown in the experimental traces (Fig. 4c) and quantified at 350 nm^48^, at 10:1, 3:1, and 1:1 ratios of oligomers to trodusquemine (Fig. 4d). Samples were also prepared under the same conditions in the absence of ANS and measured using static light scattering. Data are shown as the change in light scattering relative to oligomers in the absence of the molecule. In agreement with the turbidity measurements, we did not observe overt changes in the extent of light scattering upon the incubation of oligomers with 10:1, 3:1, and 1:1 ratios of oligomers to trodusquemine for any of the three systems (Fig. 4d). The light scattering measured for 5 µM trodusquemine in the absence of oligomers was below the detection threshold, further indicating that the small molecule is not aggregating under these conditions. In both of these measurements, the size of the oligomers was not increased at the highest concentrations of trodusquemine tested in cells (i.e. an equimolar concentration of trodusquemine, Fig. 4c, d), as was also confirmed by dynamic light scattering for αS oligomers (Supplementary Fig. 6) and high-resolution, phase controlled AFM for Aβ_40_ oligomers (Supplementary Fig. 7), suggesting that the decrease in oligomer cytotoxicity is predominantly driven by ability of trodusquemine to displace protein misfolded oligomers from cell membranes, rather than by a change in the oligomer structure induced by sub-stoichiometric to equimolar concentrations of trodusquemine. These results are furthermore consistent with our previous investigation on the effects of trodusquemine on Aβ_42_ oligomers, wherein it was observed that an equimolar concentration of the molecule did not appreciably alter the size of the oligomers^28^. Indeed, all our results indicate that displacement, rather than a change in oligomer size or hydrophobicity, is the driving force behind the suppression of oligomer toxicity in cells.

**Fig. 5:**
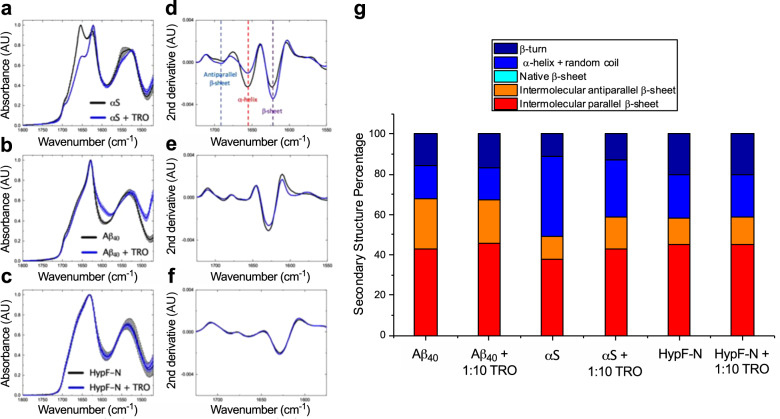
Structural characterization of the various types of oligomers with a 10-fold excess of trodusquemine. IR absorbance measurements of oligomers of αS (**a**), Aβ_40_ (**b**), and HypF-N (**c**) incubated in the absence (black) or presence of a 10-fold excess of trodusquemine (blue). The spectra were acquired in triplicate and averaged and the error bars indicate the s.e.m. of *n* = 3 replicates corresponding to independent protein depositions of the same sample. All spectra were normalized to assign an arbitrary value of 1.0 AU to the maximum absorbance. Corresponding secondary derivative analysis of the averaged spectra for αS (**d**), Aβ_40_ (**e**), and HypF-N (**f**), with key inflection points corresponding to antiparallel β-sheet (light blue), α-helix (red), and parallel β-sheet (purple) indicated with vertical dashed lines. **g** FTIR-derived secondary structure composition of the oligomers. Spectra were analyzed using a 2nd order, 12 point Savitzky-Golay filter. The presence of a 10-fold excess of trodusquemine was observed to increase the β-sheet content and reduce the combined α-helix and random coil content of αS oligomers, while the overall structural compositions of Aβ_40_ and HypF-N oligomers were largely unchanged by the presence of the molecule.

We then sought to characterize the secondary structure of the different types of oligomers in the absence and presence of trodusquemine by means of Fourier transform infrared spectroscopy (FTIR). We elected to use FTIR, as opposed to circular dichroism, given its sensitivity to detect β-sheets present in aggregated proteins, whereas circular dichroism spectroscopy can suffer from differential absorption flattening, with flattened and distorted spectra if aggregates are present in the sample^49^. Oligomers were prepared as above but at 2 mg mL^−1^, a concentration of protein that is sufficient to resolve reproducible and sufficiently intense absorbance from the oligomers, and incubated in the absence or presence of a 10-fold molar excess of trodusquemine (Fig. 5a–c). Normalized spectra were analyzed using a secondary derivative analysis (Fig. 5d–f). Even being in such excess with respect to the oligomers, the presence of trodusquemine was found to exert a negligible effect on the secondary structure of Aβ_40_ and HypF-N oligomers upon its interaction (Fig. 5g). This was similarly observed at lower concentrations of trodusquemine, wherein a 2.5-fold excess of the molecule also did not impact noticeably the secondary structure for these aggregates (Supplementary Fig. 8). It can therefore be inferred that trodusquemine does not modify the secondary structure of Aβ_40_ and HypF-N oligomers at an equimolar concentration of oligomers to trodusquemine. The αS oligomers, however, exhibited an increase in both antiparallel (+5%) and parallel (+5%) β-sheet structure with a decrease in the α-helical and random coil (−12%) content in the presence of trodusquemine (Fig. 5g). Collectively, however, these results indicate that the difference in oligomer binding to the membranes and the toxicity at and below one molar equivalent of trodusquemine (Figs. 1–3) is not likely to be related to changes in the secondary structure of the aggregates.

## Discussion

There has been a considerable focus in recent years on the soluble oligomers formed during protein aggregation, leading to the development of methods to stabilize, isolate, and characterize such species to probe the origins of their cellular toxicity and of their ability to induce synaptic dysfunction^4^. Indeed, targeting the most cytotoxic species associated with protein deposition remains an attractive strategy to develop therapeutic molecules capable of arresting the development and progression of many diseases, including Alzheimer’s, Parkinson’s and Huntington’s diseases and type 2 diabetes^1–6^. In the present study, we have probed in molecular detail the mechanism by which an aminosterol, trodusquemine, is capable of reducing the toxicity of oligomers of αS, Aβ, and HypF-N to human neuroblastoma cells.

We have found that the characteristic toxicity of these oligomers is markedly reduced when incubated in the presence of trodusquemine at concentrations at and below molar equivalence; these concentrations were able to attenuate substantially the binding affinity of oligomers to neuroblastoma cell membranes. Investigation of the effects of trodusquemine on the size of the oligomers showed that at these concentrations the size is largely unaltered relative to that in the absence of the molecule. The solvent exposed hydrophobic surface was found to be only slightly augmented at low concentrations of trodusquemine. Since the exposure of hydrophobic moieties in the interior of the oligomers has been shown previously to increase toxicity^8,45–47^, this effect would be expected to increase slightly the toxicity, but trodusquemine was found, by contrast, to protect the cells from damage initiated by these oligomeric species. Moreover, the secondary structure content of the oligomers was found to be largely maintained in the presence of even a 2.5-fold or 10-fold molar excess of the molecule. The physicochemical changes observed herein for trodusquemine are appreciably different from our recent characterization of the binding of designed antibodies to Zn^2+^-stabilized Aβ_40_ oligomers, in which we observed prominent increases in oligomer size and hydrophobicity^50^.

We therefore conclude that the dominant mechanism by which trodusquemine reduces the toxicity of misfolded protein oligomers is a displacement of the oligomers from cellular membranes rather than a change in the inherent size and hydrophobicity of the oligomers. These findings are in agreement with our previous investigations into the effects of trodusquemine on oligomers of Aβ_42_, where we found that cells pre-treated with trodusquemine prior to their exposure to oligomers experience a reduced level of oligomer binding to the cell membranes and of toxicity^28^. They are also in agreement with our past studies on the effects of the co-incubation of squalamine and trodusquemine with oligomers of αS^20,22^. Collectively with our previous studies^20,22,28^, these results demonstrate that trodusquemine can have effects also on the process of fibril formation by reducing the steady-state population of oligomers. In addition, we cannot discount completely the possibility that oligomer assembly into larger species, with reduced membrane binding and diffusional mobility, could also play a role in the in vivo mechanism of action of trodusquemine. Such a mechanism has been observed previously for a range of oligomers in the presence of a variety of molecular chaperones^30,51–53^ and some small molecules^54,55^. Moreover, evidence suggests that more subtle effects than overt physicochemical changes to oligomer structure, such as those observed upon certain chemical changes to fibrils or oligomers, may play a key role in the ability of intrinsically disordered proteins to induce toxicity^56–59^. While we have found no clear evidence of structural changes on preformed oligomers induced by the small concentrations of trodusquemine for the oligomers studied herein, as assessed by their size, surface hydrophobicity and secondary structure, we cannot exclude subtle conformational alterations.

Amphiphiles, such as aminosterols, have been suggested to be capable of partitioning into the cell membrane as a result of the presence of both charged and hydrophobic regions, where the positively charged segment of the molecule interacts with negatively charged phospholipids on the inner leaflet of the plasma membrane and hence can potentially neutralize negative surface charges on the internal part of the membrane^60–63^; indeed, such a mechanism has previously been postulated to attenuate viral replication^63^. The reduced affinity of the oligomers for cell membranes may perhaps be attributed, therefore, to the binding of trodusquemine reducing the anionic character of the lipid membranes. It is also possible that trodusquemine interacts with the membrane at regions important for oligomer binding, such as those enriched in the ganglioside GM1^37,64^. In particular, the negatively charged sialic acid of GM1 has been found to play a key role in mediating binding of the oligomers to the membrane and therefore toxicity^64^ and trodusquemine may act by reducing its charge.

Our results show that it is possible that the formation of toxic oligomers during endogenous aggregation reactions may generally be targeted directly or indirectly by trodusquemine, as the molecule can act both on the kinetics of oligomer formation and on their ability to interact with cell membranes to protect the cell from damage. These observations suggest that the protection of cell membranes by molecules such as aminosterols represents a potential route to combat the pathological effects associated with aggregation in protein misfolding diseases.

## Methods

### Preparation of HypF-N oligomers

Type A toxic oligomers were prepared as previously described^8^. Briefly, purified wild-type monomeric HypF-N was resuspended at 0.5 mg mL^−1^ in 12% (v v^−1^) TFE, 2 mM DTT, 50 mM acetate buffer, pH 5.5, and incubated at 25 °C for 4 h. The solution was centrifuged at 16,100 × *g* and 20 °C for 15 min. The supernatant was removed and residual solvent was evaporated off by gently drying the pellet with nitrogen gas, followed by resuspension in buffer (20 mM Tris, 100 mM NaCl, pH 7.4) or cell culture medium.

### Preparation of Aβ_40_ oligomers

Toxic oligomers were prepared as previously described using LoBind tubes (Eppendorf, Hamburg, Germany)^29^. Lyophilized Aβ_40_ was dissolved in 300 µL hexafluoroisopropanol (HFIP) and incubated overnight at 4 °C. The solvent was gently removed using a flow of nitrogen gas, and the film containing the protein was resuspended in 100% DMSO at a concentration of 2.2 mM. Two sonication steps of 10 min in duration were performed, after which the protein was diluted to give a final concentration of 100 µM in 20 mM sodium phosphate buffer, 200 μM ZnCl_2_, pH 6.9^29^. After 20 h, samples were centrifuged at (15 min, 16,100 × *g*, 20 °C). The supernatant was removed and the pellet containing the oligomers was resuspended to the desired peptide concentration in buffer (20 mM Tris, 100 mM NaCl, pH 7.4) or cell culture medium.

### Preparation of α-synuclein oligomers

Type B toxic oligomers of α-synuclein were prepared as previously described^33^. Briefly, the protein was purified into phosphate-buffered saline (PBS) and subsequently dialyzed against water (4 L; overnight, 4 °C). In all, 6 mg aliquots were lyophilized for 2 days, followed by resuspension in buffer (500 μL of 20 mM Tris, 100 mM NaCl, pH 7.4). The resuspended protein was passed through 0.22 μm filters and incubated (20–24 h, 37 °C). The samples were ultracentrifuged (1 h, 90,000 rpm, 20 °C) in a TLA120.2 rotor using an Optima TLX Ultracentrifuge (both Beckman Coulter, High Wycombe, UK) to remove aggregates and large oligomers. Any remaining monomeric protein was removed using a 100-kDa centrifugation filter (4×; 2 min, 10,000 rpm). The flow through containing predominantly monomeric protein from the first three passes was kept and reused up to five times. The oligomer concentration was determined by UV spectroscopy using an extinction coefficient of 5600 M^−1^cm^−1^ at 275 nm.

### Incubation of oligomers with trodusquemine

Trodusquemine was synthesized as a hydrochloride salt at a purity >97% as previously described^20,28^ and lyophilized. Aliquots at a concentration of 10 mM were prepared after dissolution in water and stored at −20 °C until use. After oligomer formation, samples were incubated in the absence or presence of trodusquemine as indicated in the text. Dynamic light scattering was used to assess if micellar or colloidal aggregates of trodusquemine were present at the highest concentration investigated in these experiments (20 mM Tris, 100 mM NaCl, pH 7.4, 50 µM trodusquemine incubated for 1 h at 20 °C). Tween 20 was measured at a concentration in excess of its critical micelle concentration as a positive control. These measurements suggest that trodusquemine is predominantly monomeric under these conditions (Supplementary Fig. 9), in agreement with our previous NMR studies^20^.

### Neuroblastoma cell culture

Human SH-SY5Y neuroblastoma cells (A.T.C.C., VA, USA) were cultured in DMEM, F-12 HAM with 25 mM HEPES and NaHCO_3_ (1:1) and supplemented with 10% FBS, 1 mM glutamine, and 1.0% antibiotics. Cell cultures were maintained in a 5% CO_2_ humidified atmosphere at 37 °C and grown until they reached 80% confluence for a maximum of 20 passages^37,64,65^. The cell lines were authenticated by the European Collection of Authenticated Cell Cultures using short tandem repeat loci analyses and they tested negatively for mycoplasma contaminations.

### MTT reduction assay

Cell viability was measured by the 3-(4,5-dimethylthiazol-2-yl)-2,5-diphenyltetrazolium bromide (MTT) reduction assay^28,37,65^. Oligomers of Aβ_40_ (5 µM, monomer equivalents) or HypF-N (6 µM, monomer equivalents) were incubated with or without increasing concentrations of trodusquemine for 1 h at 37 °C under shaking conditions, and then added to the cell culture medium of SH-SY5Y cells seeded in 96-well plates for 24 h. The molar ratios of oligomers to trodusquemine used here (monomer equivalents) were 10:1, 3:1, and 1:1. Samples were distributed throughout the mutliwell plate by random allocation. After 24 h, the cells were incubated with 0.5 mg mL^−1^ MTT at 37 °C for 4 h, and with cell lysis buffer (20% SDS, 50% *N,N*-dimethylformamide, and pH 4.7) for 3 h. The absorbance values of blue formazan that are produced upon mitochondrial reduction of MTT were determined at 590 nm. Cell viability was expressed as the percentage of MTT reduction in treated cells as compared to untreated cells.

### Oligomer binding to the cell membrane

SH-SY5Y cells were seeded on glass coverslips and treated for 15 min with HypF-N oligomers (6 µM) or Aβ_40_ oligomers (5 µM) or in the absence or presence of increasing concentrations of trodusquemine (10:1, 3:1, and 1:1 ratios of oligomers to trodusquemine, monomer equivalents). After incubation, the cells were washed with PBS and counterstained with 5.0 µg mL^−1^ Alexa Fluor 633-conjugated wheat germ agglutinin (Life Technologies, CA, USA)^22,28^. After washing with PBS, the presence of Aβ_40_ or HypF-N oligomers was detected with 1:800 diluted mouse monoclonal 6E10 anti-Aβ antibodies (BioLegend, CA, USA) or 1:800 diluted rabbit monoclonal anti-HypF-N antibodies (Primm, Milan, Italy) and subsequently with 1:1000 diluted Alexa Fluor 488-conjugated anti-mouse or anti-rabbit secondary antibodies (Life Technologies, CA, USA) to recognize the 6E10 and anti-HypF-N primary antibodies, respectively. Fluorescence emission was detected after double excitation at 488 nm and 633 nm by the scanning confocal microscopy system described previously^22^ and three apical sections were projected as a single composite image by superimposition. The percentages of oligomer colocalization were determined by analyzing regions of interest corresponding to 50–60 cells per condition.

### Model membrane measurements

All lipids were purchased from Avanti Polar Lipids, Inc. (AL, USA). Lipid vesicles comprised of DOPE:DOPS:DOPC in a ratio of 5:3:2 were prepared as previously described with sonication^44^ and at a final concentration of 100 µM. Light scattering measurements were carried out as described below.

### ANS binding measurements

In all, 8-anilinonaphthalene-1-sulfonate (ANS, Sigma-Aldrich, MO, USA) was added at a 3-fold excess with respect to oligomers (15 µM ANS to 5 µM oligomers). The binding of ANS to oligomers generates a protein-ANS complex with an increased quantum yield relative to unbound dye, where increased solvent exposure of protein-ANS complex can be observed by a blue-shift in the wavelength of maximum fluorescence and an increase in the fluorescence intensity^46^. Emission spectra were recorded in a 96-well plate (product number 3881, Corning, NY, USA) using a plate reader (BMG Labtech, Aylesbury, UK) with excitation at 380 nm. Duplicate samples are shown and the data are representative of three independent experiments using fresh oligomer preparations that gave consistent results, and all traces were background subtracted against buffer alone.

### Turbidimetry measurements

Samples from the ANS preparation were analyzed using a plate reader (BMG Labtech, Aylesbury, UK) with spectral scanning. Spectra were acquired from 350 to 650 nm and quantified according to their absorbance at 350 nm^48^. Duplicate samples are shown and the data are representative of three independent experiments using fresh oligomer preparations that gave consistent results, and all traces were background subtracted against buffer alone.

### Static light scattering

Static light scattering measurements were performed with fixed parameters for attenuator, as determined at the beginning of each series of measurements for the relevant oligomers alone sample, and cell position at 25 °C using the Malvern Zetasizer Nano S instrument (Malvern, Worcestershire, UK) equipped with a Peltier temperature controller. A low volume (70 µL) disposable cuvette was used (BRAND, Wertheim, Germany). The data are shown as the change in light scattering relative to oligomers in buffer.

### Dynamic light scattering

Dynamic light scattering was performed using the same materials and conditions described for static light scattering measurements, but with automatic settings for the attenuator and position for each sample. Measurements of trodusquemine alone were carried out at 37 °C and attenuator 11.

### Atomic force microscopy

Oligomers of Aβ_40_ were incubated at a concentration of 5 µM in the absence or presence of 5 µM trodusquemine. The mica substrate was positively functionalized by incubation with a 10-μL drop of 0.05% (v v^−1^) APTES ((3-aminopropyl)triethoxysilane, Sigma-Aldrich, MO, USA) in Milli-Q water for 1 min at ambient temperature, rinsed with Milli-Q water and then dried with a gentle flow of gaseous nitrogen^66^. AFM sample preparation was performed at room temperature by deposition of a 10-µL drop for 2 min onto the treated mica substrate. Salts were washed with water, the samples were dried by the gentle flow of nitrogen, and stored in a sealed container until imaging using a JPK Nanowizard2 AFM instrument (JPK Instruments, Berlin, Germany) with scan rates < 0.5 Hz and a silicon tip with a 10 nm nominal radius (2 N m^−1^). Three-dimensional maps were flattened using SPIP software (Image Metrology, Hørsholm, Denmark).

### Fourier transform infrared spectroscopy

Oligomeric samples were prepared at a concentration of 2 mg mL^−1^ in buffer (corresponding to monomer equivalents of 0.14 mM, 0.46 mM, and 0.18 mM for αS, Aβ_40_, and HypF-N oligomers, respectively) and incubated as above in the absence or presence of a 2.5- or 10-fold molar excess of trodusquemine. ATR-FTIR spectroscopy was performed using a Bruker Vertex 70 spectrometer equipped with a diamond ATR element (Bruker, MA, USA). Spectra were recorded with a resolution of 4 cm^−1^. Each sample was analyzed in triplicate and the spectra were averaged over 512 scans. After drying the samples by evaporation, they were washed with Milli-Q water to remove unbound trodusquemine (i.e. to minimize absorbance in the region beyond the Amide II band, see Supplementary Fig. 10 for the FTIR spectrum of trodusquemine alone) and allowed to dry prior to data collection.

### Statistics and reproducibility

Data were analyzed in GraphPad Prism 8.4 (CA, USA) by one-way ANOVA followed by Bonferroni’s post comparison test except where otherwise indicated, and *P* < 0.05 was accepted as statistically significant^28^. The *N* values, *P* values, number of technical replicates, and number of independent experiments performed for each type of measurement are listed where applicable.

### Reporting summary

Further information on research design is available in the Nature Research Reporting Summary linked to this article.

## Supplementary information

Supplementary Information

Supplementary Data 1

Reporting Summary

Peer Review File

Description of Additional Supplementary Files

## Data Availability

The data generated or analyzed in this study are included in the article and supporting information. Data for the main figures are provided as Supplementary Data 1, and all data are available from the authors upon reasonable request.
