# Supplementary material for: Trodusquemine displaces protein misfolded oligomers from cell membranes and abrogates their cytotoxicity through a generic mechanism
Source: Commun Biol. 2020 Aug 13;3:435. doi: 10.1038/s42003-020-01140-8 (PMC7426408; doi:10.1038/s42003-020-01140-8)
Supplement: Supplementary file 1 — Supplementary Information [file 42003_2020_1140_MOESM1_ESM.pdf]

Supplementary Figures

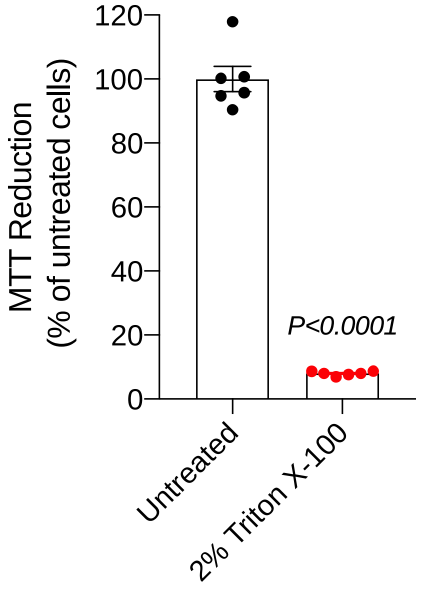

Supplementary Figure 1. Cells lysed with Triton X-100 as a positive control for the MTT assay. SH-SY5Y neuroblastoma cells were exposed to cell culture medium (black) or 2% Triton X-100 (red) for 24 h. Error bars indicate the standard error of the mean (s.e.m.) of n=6 technical replicates (dots). Conditions were compared using an unpaired, two-tailed Student’s t-test.

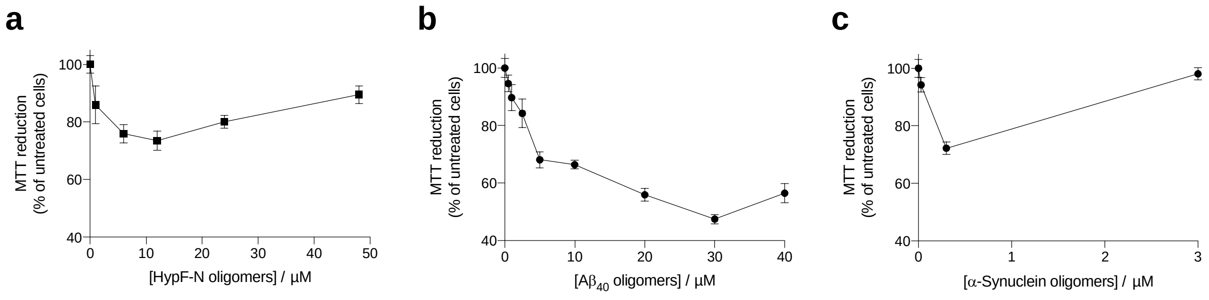

Supplementary Figure 2. Oligomer cytotoxicity to cells is reduced at low and high oligomer concentrations. Human neuroblastoma cells were incubated with 0, 1, 6, 12, 24, and 48 µM concentrations of HypF-N oligomers (in monomer equivalents) (**a**), 0, 0.5, 1, 2.5, 5, 10, 20, 30, and 40 μM concentrations of zinc-stabilized $A\beta_{40}$ oligomers (in monomer equivalents) (**b**), and 0, 0.03, 0.3, and 3 µM concentrations of αS oligomers (in monomer equivalents) (**c**). Based on these results, we tested HypF-N oligomers at a concentration of 6 µM and zinc-stabilized $A\beta_{40}$ oligomers at a concentration of 5 µM in the MTT and membrane binding measurements with and without trodusquemine. Bars indicate the mean ± s.e.m of n=3 biologically independent experiments for HypF-N and αS oligomers and the mean ± s.e.m. of n=6 technical replicates for $A\beta_{40}$ oligomers.

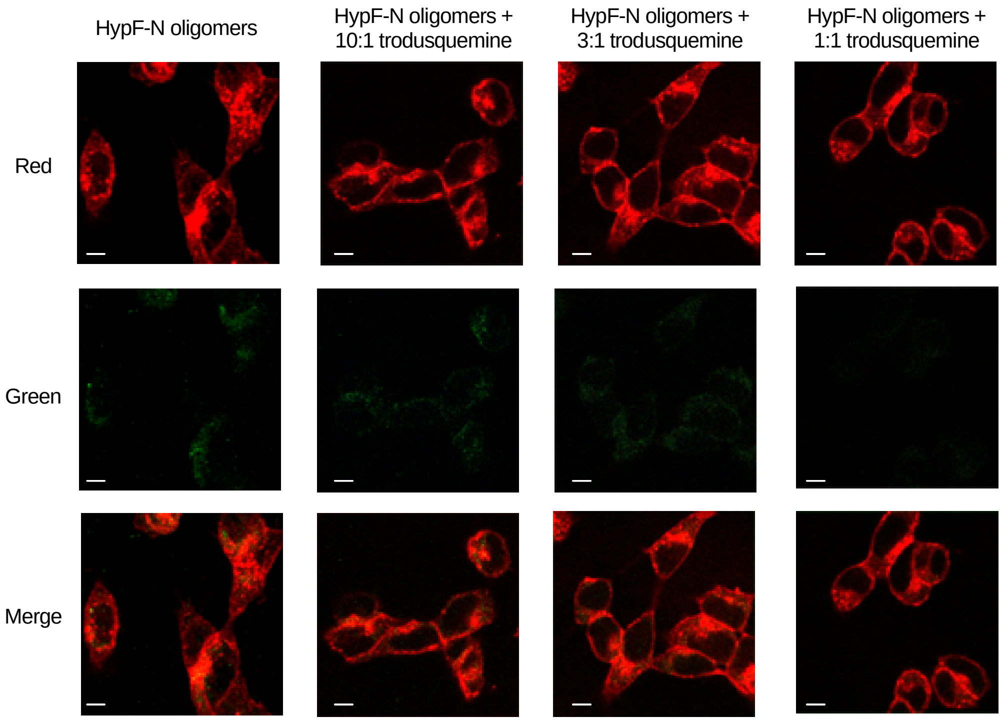

Supplementary Figure 3. Lack of HypF-N oligomer internalization into the cytosol. The median planes of the cells corresponding to Fig. 2 were analyzed to monitor the intracellular fluorescence. Red and green fluorescence indicates the cell membranes and the oligomers, respectively. Scale bars, 10 µm.

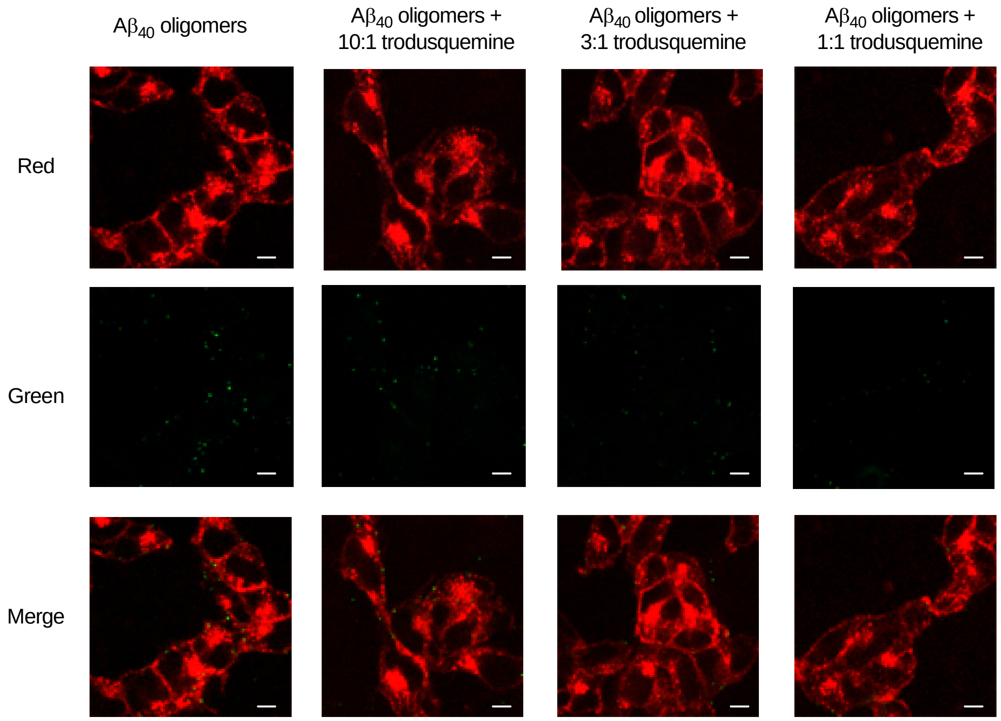

Supplementary Figure 4. Lack of Aβ_{40} oligomer internalization into the cytosol. The median planes of the cells corresponding to Fig. 3 were analyzed to monitor the intracellular fluorescence. Red and green fluorescence indicates the cell membranes and the oligomers, respectively. Scale bars, 10 µm.

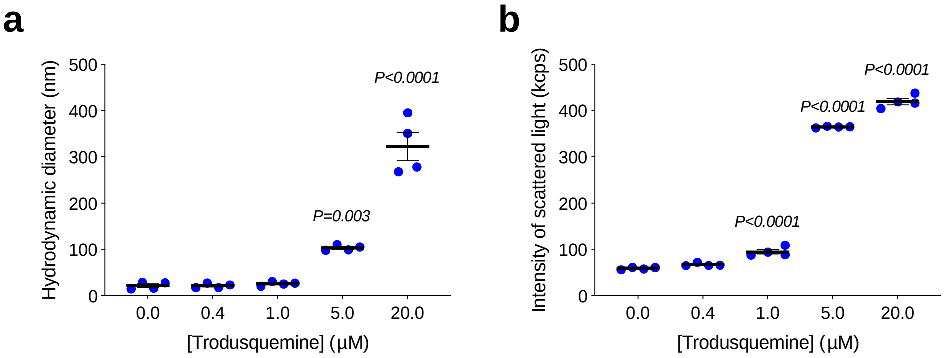

Supplementary Figure 5. Vesicle size is increased by trodusquemine. (**a**) Small unilamellar vesicles comprised of DOPE:DOPS:DOPC in a ratio of 5:3:2 at a concentration of 100 µM were incubated in the absence and presence of increasing concentrations of trodusquemine (0-20 µM). Vesicle hydrodynamic diameter was determined as the center of the distribution measured by dynamic light scattering. (**b**) Size was also monitored using static light scattering (in kilocounts per second, kcps). Data indicate mean ± s.e.m. of n=4 technical replicates.

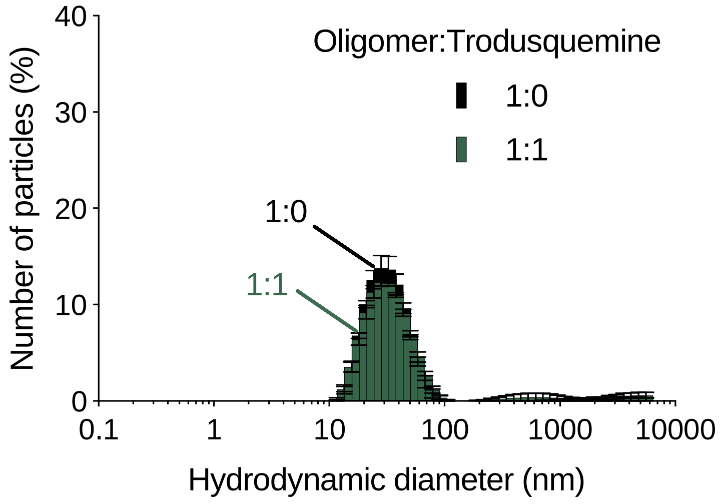

Supplementary Figure 6. Dynamic light scattering measurements for oligomers of αS incubated with trodusquemine. Oligomers of αS were incubated at a concentration of 5 µM in the absence (black) or presence of an equimolar concentration of trodusquemine (green). The presence of trodusquemine at the highest concentration tested in cells exerted a minimal effect on the size distribution of the oligomers. Bars indicate mean ± s.e.m. of n=3 technical replicates.

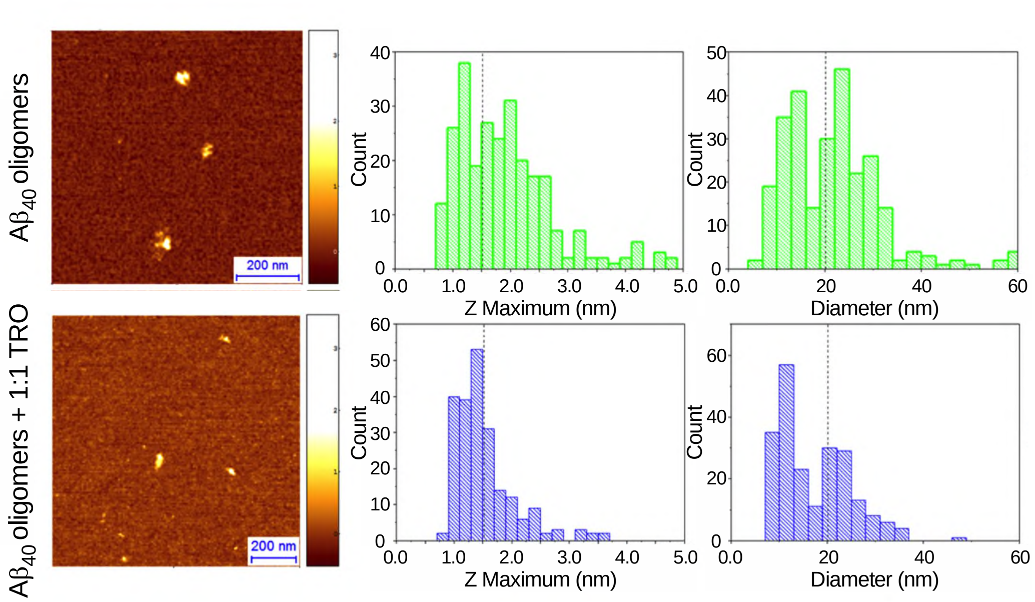

Supplementary Figure 7. Aβ_{40} oligomer size is not significantly increased in the presence of an equimolar concentration of trodusquemine. Samples were incubated as previously described in the absence (green) or presence (blue) of an equimolar ratio of trodusquemine. Oligomer deposition was performed onto a MICA substrate functionalized with APTES (see **Methods**). The maximum cross-sectional height and diameter was quantified for individual oligomers. Z range: 3.3 nm (in the color plots); the dashed vertical lines are shown at a Z maximum of 1.5 nm in the left histograms and a diameter of 20 nm in the right histograms for reference.

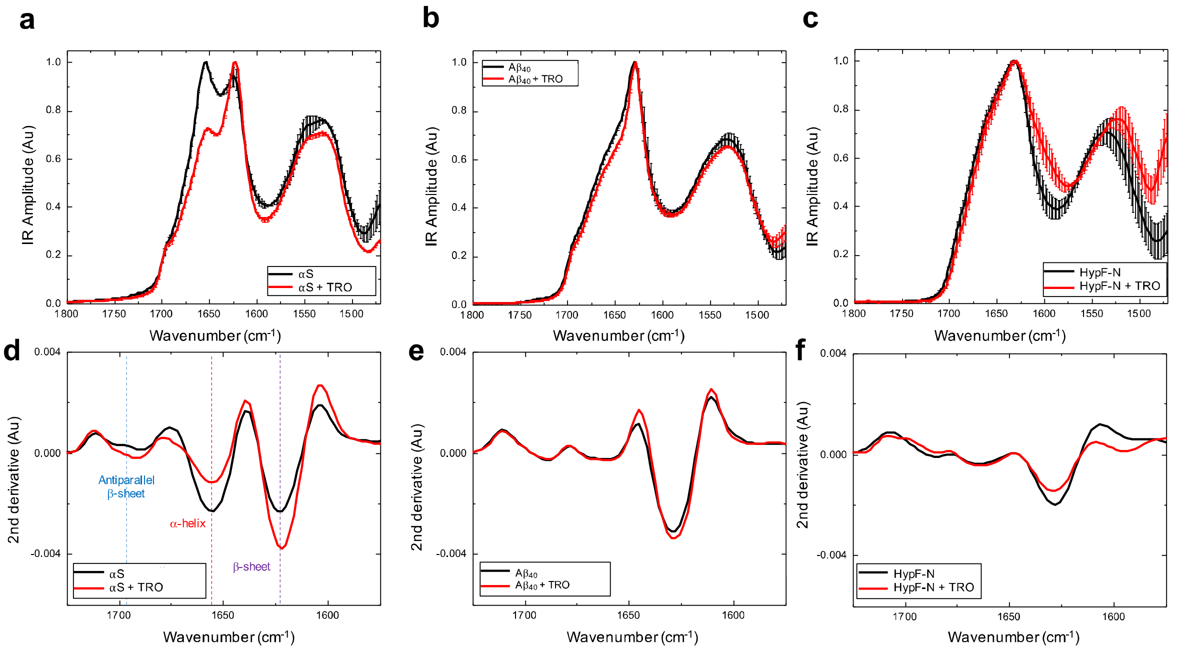

Supplementary Figure 8. Structural characterization of the various types of oligomers with a 2.5-fold excess of trodusquemine. IR absorbance measurements of oligomers of αS (**a**), $A\beta_{40}$ (**b**) and HypF-N (**c**) incubated in the absence (black traces) or presence of a 2.5-fold excess of trodusquemine (red traces). The spectra were acquired in triplicate and averaged and the error bars indicate the s.e.m. of n=3 replicates corresponding to independent protein depositions. All spectra were normalized to assign an arbitrary value of 1.0 Au to the maximum absorbance. Corresponding secondary derivative analysis of the averaged spectra for αS (**d**), $A\beta_{40}$ (**e**) and HypF-N (**f**), with key inflection points corresponding to antiparallel β-sheet (light blue), α-helix (red), and parallel β-sheet (purple) indicated with vertical dashed lines. The presence of a 2.5-fold excess of trodusquemine was observed to increase the β-sheet content and reduce the combined α-helix and random coil content of αS oligomers to a smaller extent in comparison to a 10-fold excess of trodusquemine (ref **Fig. 5**), while the overall structural compositions of $A\beta_{40}$ and HypF-N oligomers remained largely unchanged in the presence of the molecule as also observed at higher concentrations of the molecule.

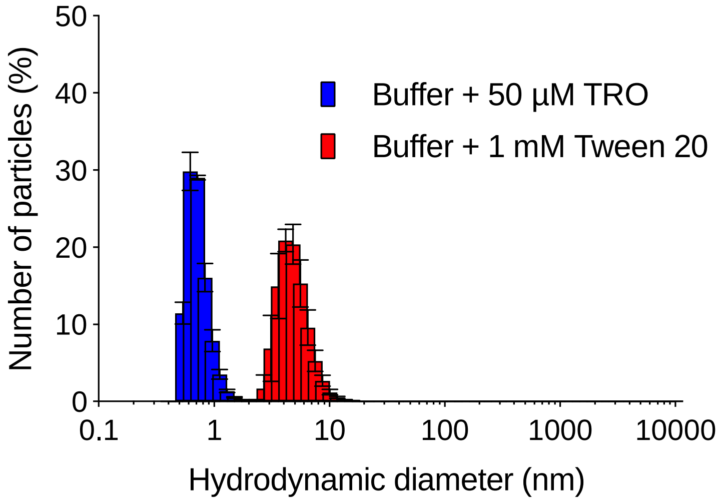

Supplementary Figure 9. Dynamic light scattering measurements of trodusquemine indicate that the molecule is predominantly monomeric. Light scattering measurements of buffer (20 mM Tris, 100 mM NaCl, pH 7.4) in the presence of 50 µM trodusquemine (blue) do not indicate the formation of micellular or colloidal aggregates of the molecule. Tween 20 (red) was measured at a concentration in excess of its critical micelle concentration as a positive control. Bars indicate mean ± s.e.m. of n=3 technical replicates.

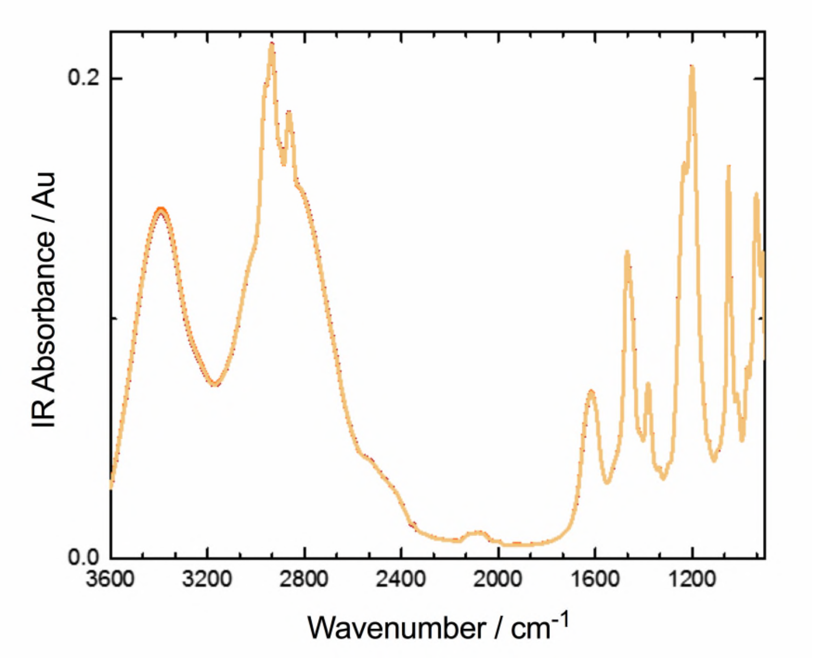

Supplementary Figure 10. FTIR spectrum of trodusquemine. A spectrum of 10 mM trodusquemine was measured in the absence of oligomers. Care was taken to ensure unbound molecules were washed away in the presence of oligomers, as assessed by the disappearance of the molecule absorbance peaks not related to the signal from the protein.
