# Supplementary material for: Trodusquemine displaces protein misfolded oligomers from cell membranes and abrogates their cytotoxicity through a generic mechanism
Source: Commun Biol. 2020 Aug 13;3:435. doi: 10.1038/s42003-020-01140-8 (PMC7426408; doi:10.1038/s42003-020-01140-8)
Supplement: Supplementary file 3 — Reporting Summary [file 42003_2020_1140_MOESM3_ESM.pdf]

nature research

Corresponding author(s): Fabrizio Chiti and Michele Vendruscolo

Last updated by author(s): Jun 22, 2020

Reporting Summary

Nature Research wishes to improve the reproducibility of the work that we publish. This form provides structure for consistency and transparency in reporting. For further information on Nature Research policies, see Authors & Referees and the Editorial Policy Checklist.

Statistics

For all statistical analyses, confirm that the following items are present in the figure legend, table legend, main text, or Methods section.

n/a	Confirmed	
☐	☒	The exact sample size (n) for each experimental group/condition, given as a discrete number and unit of measurement
☐	☒	A statement on whether measurements were taken from distinct samples or whether the same sample was measured repeatedly
☐	☒	The statistical test(s) used AND whether they are one- or two-sided<br>*Only common tests should be described solely by name; describe more complex techniques in the Methods section.*
☒	☐	A description of all covariates tested
☐	☒	A description of any assumptions or corrections, such as tests of normality and adjustment for multiple comparisons
☐	☒	A full description of the statistical parameters including central tendency (e.g. means) or other basic estimates (e.g. regression coefficient) AND variation (e.g. standard deviation) or associated estimates of uncertainty (e.g. confidence intervals)
☐	☒	For null hypothesis testing, the test statistic (e.g. F, t, r) with confidence intervals, effect sizes, degrees of freedom and P value noted<br>*Give P values as exact values whenever suitable.*
☒	☐	For Bayesian analysis, information on the choice of priors and Markov chain Monte Carlo settings
☒	☐	For hierarchical and complex designs, identification of the appropriate level for tests and full reporting of outcomes
☒	☐	Estimates of effect sizes (e.g. Cohen's d, Pearson's r), indicating how they were calculated

Our web collection on statistics for biologists contains articles on many of the points above.

Software and code

Policy information about availability of computer code

Data collection	No specialized software used.
Data analysis	ImageJ was used for confocal microscopy image analysis. Statistical tests were carried out as described in the manuscript using GraphPad Prism 8.4.

For manuscripts utilizing custom algorithms or software that are central to the research but not yet described in published literature, software must be made available to editors/reviewers. We strongly encourage code deposition in a community repository (e.g. GitHub). See the Nature Research guidelines for submitting code & software for further information.

Data

Policy information about availability of data

All manuscripts must include a data availability statement. This statement should provide the following information, where applicable:

- Accession codes, unique identifiers, or web links for publicly available datasets
- A list of figures that have associated raw data
- A description of any restrictions on data availability

The data generated or analyzed in this study are included in the article and supporting information. Data for the main figures are provided as Supplementary Data 1, and all data are available from the authors upon reasonable request.

Field-specific reporting

Please select the one below that is the best fit for your research. If you are not sure, read the appropriate sections before making your selection.

☒ Life sciences ☐ Behavioural & social sciences ☐ Ecological, evolutionary & environmental sciences

For a reference copy of the document with all sections, see nature.com/documents/nr-reporting-summary-flat.pdf

Life sciences study design

All studies must disclose on these points even when the disclosure is negative.

Sample size	Sample sizes for the SH-SY5Y experiments were determined based on previous studies (Perni et al., PNAS, 2017; Perni et al., ACS Chem. Biol., 2018; Limbocker et al., Nature Communications, 2019).
Data exclusions	No data were excluded from the study.
Replication	Experiments were carried out with the number of replicates indicated in the manuscript. All attempts at replication were successful.
Randomization	Samples were distributed throughout the mutliwell plate using a random allocation approach for the MTT tests. Randomization was not necessary for the other experiments.
Blinding	No blinding.

Reporting for specific materials, systems and methods

We require information from authors about some types of materials, experimental systems and methods used in many studies. Here, indicate whether each material, system or method listed is relevant to your study. If you are not sure if a list item applies to your research, read the appropriate section before selecting a response.

Materials & experimental systems

n/a	Involved in the study	
☐	☒	Antibodies
☐	☒	Eukaryotic cell lines
☒	☐	Palaeontology
☒	☐	Animals and other organisms
☒	☐	Human research participants
☒	☐	Clinical data

Methods

n/a	Involved in the study	
☒	☐	ChIP-seq
☒	☐	Flow cytometry
☒	☐	MRI-based neuroimaging

Antibodies

Antibodies used	monoclonal anti-amyloid-beta antibody (anti-mouse, clone 6E10, BioLegend, CA, USA)<br>polyclonal anti-HypF-N antibody (anti-rabbit, Primm, Milan, Italy)<br>Alexa 488-conjugated secondary antibody (anti-mouse, Life Technologies, CA, USA)<br>Alexa 488-conjugated secondary antibody (anti-rabbit, Life Technologies, CA, USA)
Validation	Antibodies were used and stored according to the manufacturer's instructions.

Eukaryotic cell lines

Policy information about cell lines

Cell line source(s)	SH-SY5Y human neuroblastoma cells were acquired from Sigma-Aldrich, MO, USA (origin from A.T.C.C. (VA, USA)).
Authentication	The cell line was authenticated by the European Collection of Authenticated Cell Cultures using short tandem repeat loci analyses.
Mycoplasma contamination	The cell line tested negative for mycoplasma contaminations.
Commonly misidentified lines<br>(See ICLAC register)	Not applicable.
