# Supplementary material for: Trodusquemine displaces protein misfolded oligomers from cell membranes and abrogates their cytotoxicity through a generic mechanism
Source: Commun Biol. 2020 Aug 13;3:435. doi: 10.1038/s42003-020-01140-8 (PMC7426408; doi:10.1038/s42003-020-01140-8)
Supplement: Supplementary file 5 — Description of Additional Supplementary Files [file 42003_2020_1140_MOESM5_ESM.pdf]

Description of Additional Supplementary Files

File Name: **Supplementary Data 1**
Description: MTT, oligomer binding, ANS, turbidity, SLS, and FTIR data.
