# Supplementary material for: Trodusquemine displaces protein misfolded oligomers from cell membranes and abrogates their cytotoxicity through a generic mechanism
Source: Commun Biol. 2020 Aug 13;3:435. doi: 10.1038/s42003-020-01140-8 (PMC7426408; doi:10.1038/s42003-020-01140-8)
Supplement: Supplementary file 4 — Peer Review File [file 42003_2020_1140_MOESM4_ESM.pdf]

Reviewers' comments:

Reviewer #1 (Remarks to the Author):

This is an interesting manuscript, which I would be happy to recommend for publication, once the following concerns have been addressed:

1. The oligomeric preparations shown in Figure 1 are not particularly toxic, as they reduce cell viability by about 25-30 %. The authors should conduct experiments at either higher concentration or use more toxic peptide, e.g. using Aβ42 in addition to Aβ40.

2. There is mounting evidence that uptake of Aβ is a key step towards many of its toxic actions. The authors should discuss that by reducing affinity of their peptides for membranes they are suppressing uptake of their IDPs studied, and that this may be the underlying reason, or, at least a major contributor why toxicity is reduced. The following papers should be discussed:
a) S. Jin, N. Kedia, E. Illes-Toth, I. Haralampiev, S. Prisner, A. Herrmann, E. E. Wanker, J. Bieschke, J. Biol. Chem. 2016, 291, 19590–19606
b) S. Dutta, A. R. Foley, C. J. A. Warner, X. Zhang, M. Rolandi, B. Abrams, J. A. Raskatov, Angew. Chem. Int. Ed. 2017, 56, 11506–11510
c) Dutta, S., Finn, T. S., Kuhn, A. J., Abrams, B., Raskatov, J. A. ChemBioChem 2019, 20, 1023−1026.

3. The authors present compelling evidence that oligomer size distribution is not changed upon treatment with trodusquemine. However, there is mounting evidence that more subtle effects may underlie toxic actions of IDPs, e.g., the recently introduced concept of the toxic turn. The authors should augment their discussion with the following papers and, if possible, provide molecular level information on what happens to the oligomers upon addition of trodusquemine. NMR might work, but the experiments may not be straightforward enough to be included into this study. If this turns out to be the case, the authors might wish to consider it for follow-up work.

a) Y. Matsushima, R. C. Yanagita and K. Irie, Chem. Commun., 2020, DOI: 10.1039/D0CC01053G.
b) C. J. A. Warner, S. Dutta, A. R. Foley and J. A. Raskatov, Chem. Eur. J., 2016, 22, 11967–11970.
c) A. R. Foley, T. S. Finn, T. Kung, A. Hatami, H.-W. Lee, M. Jia, M. Rolandi, J. A. Raskatov, ACS Chem. Neurosci., 2019, 10, 3880–3887.
d) A. R. Foley, H.-W. Lee,J. A. Raskatov, J. Org. Chem., 2020, 85, 1385–1391.

Once these points have been addressed, I will be happy to recommend this manuscript for publication. Alternatively, the manuscript could be published without further experiments (i.e., omitting #1) in Scientific Reports, just by augmenting the discussion.

Reviewer #2 (Remarks to the Author):

The manuscript describes a mechanistic study of the protective effect of trodusquemine, an aminosterol, against the toxicity in cell culture of oligomers of HypF-N and Aβ40 bound to Zn^{2+}. The compound previously has been shown to be protective also against Aβ42 and α-synuclein oligomers. Here, the authors examine the effect of the compound on membrane binding of the oligomers of HypF-N, Aβ40-Zn^{2+}, and α-synuclein and on the structure of the oligomers themselves. Based on their analysis, the authors suggest that inhibition of membrane binding is the predominant mechanism by which trodusquemine exerts its protective effect. This conclusion is important for development of

compounds targeting oligomers in proteinopathies, yet it is only partially supported by the data due to issues with the experimental design and data presentation as listed below:

Major issues

1. The choice of Aβ40 oligomers stabilized by Zn^{2+} is unusual and no explanation or justification is given for this choice. Are these oligomers expected to represent those in the AD brain?
2. As the authors are well aware, because oligomers are metastable structures, obtaining them reproducibly is challenging. Although each type of oligomer has been obtained using a published protocol, some form of validation of the integrity of these structures should be presented.
3. The previous studies by these authors suggested that trodusquemine inhibits α-synuclein aggregation and promotes Aβ42 aggregation. How can these data be reconciled with the conclusion that the compound has a minimal effect on oligomer structure?
4. If the conclusion of the manuscript is correct, how would it affect the normal association of α-synuclein with cell membranes in vivo? Interference with the binding of α-synuclein to synaptic vesicles may have a detrimental, rather than a therapeutic effect.
5. A positive control is missing in the MTT assay (full killing of the cells).
6. In all the bar graphs in the different figures, please include the individual data points and provide actual p-values, rather than the arbitrary cutoffs at 0.05, 0.01 etc.
7. It is not clear why in most of the images in Figures 2 and 3 the green and red do not overlap. Do the oligomers bind to the membrane in regions not labeled by wheat-germ agglutinin?
8. The data shown in figures 2 and 3 suggest that the impact of trodusquemine on the binding of HypF-N oligomers to the cells is stronger than on the binding of Aβ40-Zn^{2+} oligomers. The images disagree with the quantitative data, especially when the compound is applied at an equimolar concentration.
9. If the mechanism by which trodusquemine interfere with oligomer binding is by increasing the rigidity of the cell membrane, a correlation between rigidity and degree of inhibition would be expected. Can such a correlation be tested by examining a few other aminosterols? Would compounds that decrease membrane rigidity increase oligomer binding?
10. In Figure 4d, the increase in the static light scattering of HypF-N oligomers in the presence of different concentrations of trodusquemine appears to be substantial and consistent at all concentrations compared to HypF-N oligomers in the absence of trodusquemine. The conclusion that the compound has no effect does not appear to be supported by the data.
11. The interpretation proposed in lines 245-246, "…the decrease in oligomer cytotoxicity is not mediated by a change in the oligomer structure…" is going too far. Later in the Discussion, the authors acknowledge that changes in oligomer structure may contribute to the changes in toxicity, though disruption of the interaction with the membrane may be the main mechanism. This interpretation is in line with the data, but not the sentence cited above.
12. DLS data are presented only for α-synuclein and AFM only for Aβ. These analyses should be done and presented for all three proteins.
13. The AFM analysis of Aβ shows clearly that the oligomer size distribution is shifted toward smaller ones in the presence of trodusquemine, in contrast to the claims in the text.
14. FTIR is not a very sensitive method for measuring small changes in secondary structure of amyloid proteins. Adding CD analysis will be more convincing. In addition, the protein concentration used in the FTIR and the large excess of trodusquemine make it impractical to correlate these data with other experiments. The same concentration ratios should be used as in all other experiments.
15. The hypothesis that charge contributes to the effect of trodusquemine, particularly "the negatively charged sialic acid of GM1" (lines 315-316) is interesting. Can it be tested experimentally?

Minor issues
1. WHO-mandated nomenclature is that Aβ is an abbreviation of amyloid β-protein, not amyloid-β

peptide.
2. In line 219 the concentration of trodusqumine is described as “0.1, 0.33, and 1 molar equivalents” after in all previous instances it was described as “10:1, 3:1, and 1:1 ratios”. For clarity, it would be better to use one consistent description.
3. The concentration units used for the IR experiments, mg/mL, are inconsistent with all the other cases, where the concentration is given in μL. The authors should use consistent units throughout the manuscript.
4. References to Figures 5A and 5B are missing in the text.
5. In the sentence starting with “After washing with PBS…” in line 398, the mention of HypF-N oligomers is missing.
6. The grammar of the first sentence in the ANS binding measurements (line 414) should be corrected.
7. The “Light scattering” section (starting at line 432) is confusing. It starts with SLS and continues with DLS. Separate sections should be included for the two techniques.

Author Reply (point by point basis)

We thank the reviewers and the *Communications Biology* editorial team for their positive feedback and careful analysis of our work. We have now updated our manuscript to address the points raised by the reviewers, and in doing so appreciate that our presentation has been significantly improved thanks to their very helpful comments.

With these revisions, we hope that the manuscript will now meet the standard for publication in *Communications Biology.*

In our point-by-point response that follows, reviewers' comments are shown in black, and our answers in blue. All corresponding changes to the manuscript and supplementary information have been highlighted in the revised manuscript.

Reviewer #1 (Remarks to the Author):

This is an interesting manuscript, which I would be happy to recommend for publication, once the following concerns have been addressed:

We thank the reviewer for his/her positive comments on our work.

1. The oligomeric preparations shown in Figure 1 are not particularly toxic, as they reduce cell viability by about 25-30 %. The authors should conduct experiments at either higher concentration or use more toxic peptide, e.g. using Aβ42 in addition to Aβ40.

We thank the reviewer for this insightful suggestion. To address this important issue, we are now reporting new experiments showing the effect of increasing oligomer concentration towards the viability of human neuroblastoma cells. The following paragraph has been added to the results section of the manuscript (**lines 192-202** in the revised version):

The oligomer concentrations administered to cells of 6 µM, 5 µM, and 0.3 µM for HypF-N, zinc-stabilized Aβ40 and αS oligomers, respectively, were selected based on preliminary observations that oligomer toxicity peaks at these concentrations. To illustrate this point, we incubated human neuroblastoma cells with 0, 1, 6, 12, 24, and 48 µM concentrations of HypF-N oligomers, 0, 0.5, 1, 2.5, 5, 10, 20, 30, and 40 µM concentrations of zinc-stabilized Aβ40 oligomers, and 0, 0.03, 0.3, and 3 µM concentrations of αS oligomers (Supplementary Figure 2). For these three systems, we found that the toxicity correlated non-linearly with the oligomer concentration, given that at high concentrations oligomers coalescence into larger, less toxic aggregates. Also in consideration of the observation that trodusquemine is soluble and monomeric at a concentration of 10 µM in phosphate buffered saline (Perni et al., ACS Chem. Biol., 2018), we therefore elected to use the aforementioned concentrations of oligomers ex vivo and in vitro.

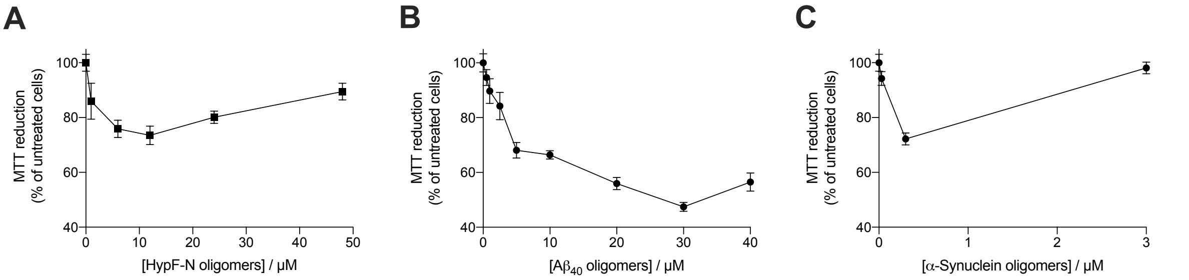

Supplementary Figure 2. Oligomer cytotoxicity to cells is reduced at low and high oligomer concentrations. *Human neuroblastoma cells were incubated with 0, 1, 6, 12, 24, and 48 µM concentrations of HypF-N oligomers (in monomer equivalents) (**a**), 0, 0.5, 1, 2.5, 5, 10, 20, 30, and 40 µM concentrations of zinc-stabilized Aβ$_{40}$ oligomers (in monomer equivalents) (**b**), and 0, 0.03, 0.3, and 3 µM concentrations of αS oligomers (in monomer equivalents) (**c**). Based on these results, we tested HypF-N oligomers at a concentration of 6 µM and zinc-stabilized Aβ$_{40}$ oligomers at a concentration of 5 µM in the MTT and membrane binding measurements with and without trodusquemine. Bars indicate the mean ± s.e.m of three independent experiments.*

We have previously studied the effects of trodusquemine on isolated Aβ$_{42}$ oligomers (Limbocker et al., *Nat. Commun.*, 2019). In that work, we investigated a concentration of 1 µM of oligomers. As the reviewer correctly noted, those oligomers were indeed more toxic than the zinc-stabilized Aβ$_{40}$ oligomers used in the present work when corrected for concentration, which is consistent with previous observations regarding the aggregation propensity and hydrophobicity differences between these two protein fragments (Meisl et al., *PNAS*, 2014). We have updated the manuscript to address these points (**lines 164-166** in the revised version), Other studies have similarly illustrated that increasing the concentration of this Aβ$_{42}$ oligomer model also does not correlate linearly to increased toxicity (in particular Figure 6b of Ming-Che Lee et al., *Sci. Rep.*, 2018 where they employed ADDLs by Aβ$_{42}$ from 10 to 40 µM).

2. There is mounting evidence that uptake of Aβ is a key step towards many of its toxic actions. The authors should discuss that by reducing affinity of their peptides for membranes they are suppressing uptake of their IDPs studied, and that this may be the underlying reason, or, at least a major contributor why toxicity is reduced. The following papers should be discussed:
a) S. Jin, N. Kedia, E. Illes-Toth, I. Haralampiev, S. Prisner, A. Herrmann, E. E. Wanker, J. Bieschke, J. Biol. Chem. 2016, 291, 19590–19606
b) S. Dutta, A. R. Foley, C. J. A. Warner, X. Zhang, M. Rolandi, B. Abrams, J. A. Raskatov, Angew. Chem. Int. Ed. 2017, 56, 11506–11510
c) Dutta, S., Finn, T. S., Kuhn, A. J., Abrams, B., Raskatov, J. A. ChemBioChem 2019, 20, 1023−1026.

The reviewer correctly notes that the displacement of Aβ oligomers, in addition to oligomers of HypF-N and αS, by the common mechanism displayed by trodusquemine could also result in differential Aβ uptake, which has been described in the above studies to play an important role in mediating cytotoxicity. To provide better context to our work, we have referenced those articles and added an analysis of the medial planes of the cells with the following explanation (**lines 233-239** of the revised text):

*Recent studies have demonstrated that the uptake of intrinsically disorder proteins, including Aβ, is a key step in its mechanism of toxicity (Jin et al, J. Biol. Chem., 2016; Dutta et al., Angew. Chem. Int. Ed., 2017; Dutta et al., ChemBioChem, 2019). It is therefore possible that the trodusquemine-induced displacement of oligomers from cell membranes could also suppress protein uptake and its associated toxicity, as the binding of the oligomers to the membrane is a necessary step for their internalization. Indeed, we observed minimal oligomer internalization in the presence of trodusquemine, when the cells were analyzed at median planes to focus on their interior (**Supplementary Figures 3 and 4**).*

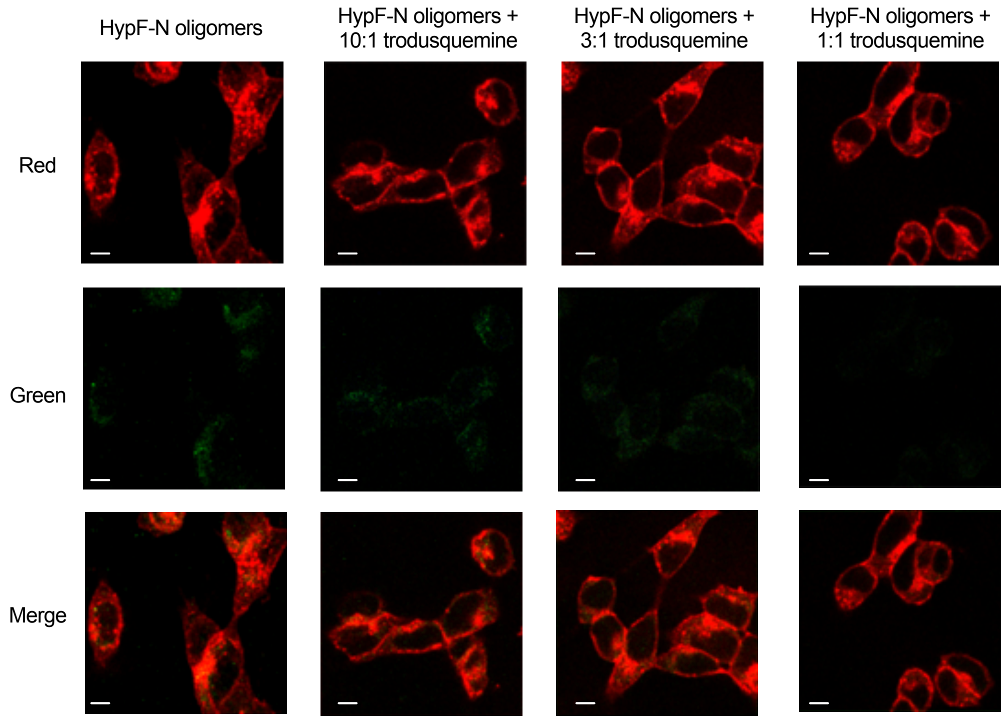

***Supplementary Figure 3. Lack of HypF-N oligomer internalization into the cytosol.** The median planes of the cells corresponding to Fig. 2 were analyzed to monitor the intracellular fluorescence. Red and green fluorescence indicates the cell membranes and the oligomers, respectively. Scale bars, 10 µm.*

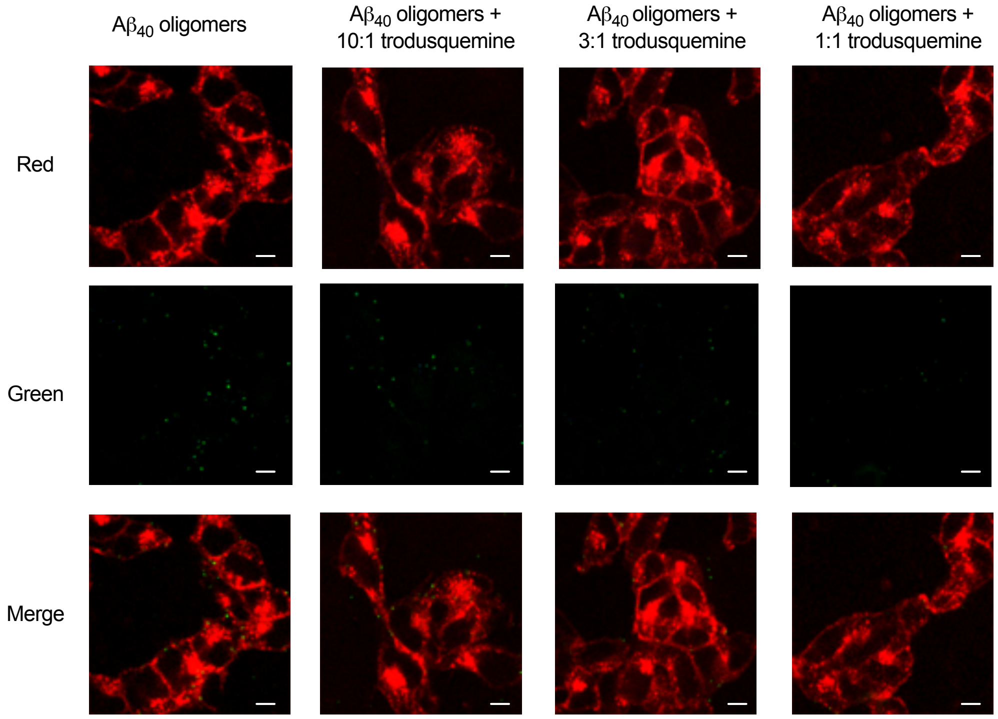

Supplementary Figure 4. Lack of Aβ_{40} oligomer internalization into the cytosol. *The median planes of the cells corresponding to Fig. 3 were analyzed to monitor the intracellular fluorescence. Red and green fluorescence indicates the cell membranes and the oligomers, respectively. Scale bars, 10 μm.*

3. The authors present compelling evidence that oligomer size distribution is not changed upon treatment with trodusquemine. However, there is mounting evidence that more subtle effects may underlie toxic actions of IDPs, e.g., the recently introduced concept of the toxic turn. The authors should augment their discussion with the following papers and, if possible, provide molecular level information on what happens to the oligomers upon addition of trodusquemine. NMR might work, but the experiments may not be straightforward enough to be included into this study. If this turns out to be the case, the authors might wish to consider it for follow-up work.

a) Y. Matsushima, R. C. Yanagita and K. Irie, Chem. Commun., 2020, DOI: 10.1039/D0CC01053G.
b) C. J. A. Warner, S. Dutta, A. R. Foley and J. A. Raskatov, Chem. Eur. J., 2016, 22, 11967–11970.
c) A. R. Foley, T. S. Finn, T. Kung, A. Hatami, H.-W. Lee, M. Jia, M. Rolandi, J. A. Raskatov, ACS Chem. Neurosci., 2019, 10, 3880–3887.
d) A. R. Foley, H.-W. Lee,J. A. Raskatov, J. Org. Chem., 2020, 85, 1385–1391.

The reviewer brings up a stimulating point. We have added these references and a discussion (lines **365-371** in the revised text) to address it:

Moreover, evidence suggests that more subtle effects than overt biophysical changes to oligomer structure, such as those observed upon certain chemical changes to fibrils or oligomers, may play a significant role in the ability of intrinsically disordered proteins to

induce toxicity (Matsushima et al., Chem. Commun., 2020; Warner et al., Chem. Eur. J., 2016; Foley et al., ACS Chem. Neuro., 2019; Foley et al., J. Org. Chem., 2020). While we have found no evidence of structural changes on preformed oligomers induced by small concentrations of trodusquemine for the oligomers studied herein, as assessed by their size, ANS binding and secondary structure, we cannot exclude subtle conformational alterations.

Once these points have been addressed, I will be happy to recommend this manuscript for publication. Alternatively, the manuscript could be published without further experiments (i.e., omitting #1) in Scientific Reports, just by augmenting the discussion.

We thank the reviewer for his/her constructive feedback, and appreciate that the quality of our presentation has improved significantly thanks to his/her efforts.

Reviewer #2 (Remarks to the Author):

The manuscript describes a mechanistic study of the protective effect of trodusquemine, an aminosterol, against the toxicity in cell culture of oligomers of HypF-N and Aβ40 bound to Zn2+. The compound previously has been shown to be protective also against Aβ42 and α-synuclein oligomers. Here, the authors examine the effect of the compound on membrane binding of the oligomers of HypF-N, Aβ40-Zn2+, and α-synuclein and on the structure of the oligomers themselves. Based on their analysis, the authors suggest that inhibition of membrane binding is the predominant mechanism by which trodusquemine exerts its protective effect. This conclusion is important for development of compounds targeting oligomers in proteinopathies, yet it is only partially supported by the data due to issues with the experimental design and data presentation as listed below:

We thank the reviewer his/her careful analysis of our work and for highlighting the importance of the conclusions of our study towards developing compounds against oligomers in a wide range of human proteinopathies.

Major issues

1. The choice of Aβ40 oligomers stabilized by Zn2+ is unusual and no explanation or justification is given for this choice. Are these oligomers expected to represent those in the AD brain?

We thank the reviewer for highlighting this important aspect of our presentation. We have now added the following to **lines 159-164** of the revised text to motivate our choice:

Aβ_{40} oligomers stabilized by zinc represent a valuable model for studying the acute oligomer toxicity associated with AD because they are not stabilized by an invasive chemical-physical modification, but rather by a metabolite that is naturally present at synapses. This model can therefore resemble populations of oligomers endogenously occurring in the brain (Mannini et al., ACS Chem. Neuro., 2018).

2. As the authors are well aware, because oligomers are metastable structures, obtaining them reproducibly is challenging. Although each type of oligomer has been obtained using a published protocol, some form of validation of the integrity of these structures should be presented.

We are grateful to the reviewer for raising this key point. The protocols for obtaining the oligomers used throughout our manuscript have been published and described in detail (Campioni et al., *Nat. Chem. Biol.*, 2010; Mannini et al., *ACS Chem. Neuro.*,2018; Chen et al., *PNAS*, 2015), as have their structures. Some of the authors of the present manuscript are the same that have set up these protocols, which are used routinely in their labs. A key advantage of these methods for stabilizing oligomers is that they are reproducible and yield markedly more homogeneous and stable populations.

We have now updated the manuscript to more clearly highlight this advantage (lines **133-140** of the revised text). To this point, we cite at least four papers in the manuscript for each oligomer type. With respect to the quality of our oligomer preparations and how they compare to our previous studies, we have validated their structures and biological activities herein extensively in the absence and presence of trodusquemine using FTIR, ANS, sizing by solution-based and single-molecule based methods, toxicity assays, and membrane binding assays.

3. The previous studies by these authors suggested that trodusquemine inhibits α-synuclein aggregation and promotes Aβ42 aggregation. How can these data be reconciled with the conclusion that the compound has a minimal effect on oligomer structure?

Our previous studies of trodusquemine (Limbocker et al., *Nat. Commun.*, 2019 and Perni et al., *ACS Chem. Biol.*, 2018) resolve the effects of this molecule on amyloid formation from the monomeric state, and not on its effects on performed oligomers as carried out herein. Despite the distinct mechanisms of the aggregation of αS and $A\beta_{42}$ and the differing effects of trodusquemine on the underlying processes, these effects resulted in a lower steady-state concentration of oligomers in both cases (Limbocker et al., *Nat. Commun.*, 2019). Indeed, in the case of $A\beta_{42}$ the trodusquemine-mediate acceleration of the secondary nucleation step promotes the conversion of low molecular weight species into fibrils, therefore reducing the concentration of harmful species. We have updated the manuscript accordingly to address this important point (**lines 93-97 and 357-360** of the revised text).

4. If the conclusion of the manuscript is correct, how would it affect the normal association of α-synuclein with cell membranes in vivo? Interference with the binding of α-synuclein to synaptic vesicles may have a detrimental, rather than a therapeutic effect.

The reviewer identifies an important point. In Fig. S2C of Perni et al., *ACS Chem. Biol.*, 2018, we provided clear evidence that trodusquemine does not displace monomeric α-synuclein from the membrane of DMPS vesicles at the low concentrations used for the toxicity studies (0.3 μM or so). By contrast, it displaces preformed oligomers (Fig. 2C of Perni et al., *ACS Chem. Biol.*, 2018). These data indicate that physiologically relevant concentrations of trodusquemine do not impact the normal association of α-synuclein with cell membranes. We have addressed these relevant points in the revised manuscript (lines **184-187** of the revised version), with thanks to the reviewer.

5. A positive control is missing in the MTT assay (full killing of the cells).

We have added the requested positive control as **Supplementary Figure 1** with a corresponding addition to the results (**lines 155-157** of the revised manuscript).

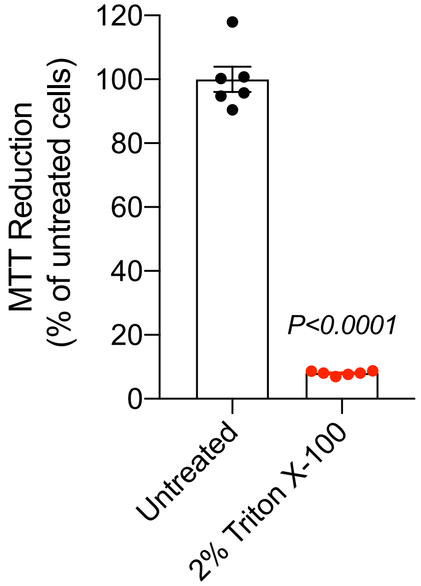

Supplementary Figure 1. Cells lysed with Triton X-100 as a positive control for the MTT assay. *SH-SY5Y neuroblastoma cells were exposed to cell culture medium (black) or 2% Triton X-100 (red) for 24 h. Error bars indicate the standard error of the mean (s.e.m.) of the six indicated technical replicates (dots). Conditions were compared using an unpaired, two-tailed Student's t-test.*

6. In all the bar graphs in the different figures, please include the individual data points and provide actual p-values, rather than the arbitrary cutoffs at 0.05, 0.01 etc.

We have updated the figures and legends accordingly, with thanks to the reviewer for this suggestion that enhances the clarity of the manuscript. Changes are shown in Figures 1, 2, 3, and 4 and Supplementary Figures 1 and 5 of the revised version.

7. It is not clear why in most of the images in Figures 2 and 3 the green and red do not overlap. Do the oligomers bind to the membrane in regions not labeled by wheat-germ agglutinin?

In Figures 2 and 3, we showed the colocalization of the oligomers with the cellular membrane in regions of interest by using ImageJ software (National Institutes of Health, Bethesda, MD, USA). In order to quantify the degrees of co-localization of oligomers with the cell membranes by the Pearson's Correlation Coefficient, we selectively analyze the oligomers bound to the cellular membrane and exclude any intracellular signals of internalized oligomers. Thus, we have selected just three confocal sections in the xy planes at the apical positions and projected them as a single composite image by superimposition in Figures 2 and 3. The presence of oligomers (small green dots) in regions where red fluorescence of wheat-germ agglutinin is missing can be ascribed to a very few proportion of internalized oligomers. The

colocalization of the two channels in Figures 2 and 3 is appropriate and consistent with previous publications (Campioni et al., *Nat. Chem. Biol.*, 2010; Perni et al., *PNAS*, 2017; Perni et al., *ACS Chem. Biol.*, 2018; Limbocker et al., *Nat. Commun*., 2019; Cascella et al., *IJMS*, 2020; Fusco et al., *Science*, 2017; etc.).

8. The data shown in figures 2 and 3 suggest that the impact of trodusquemine on the binding of HypF-N oligomers to the cells is stronger than on the binding of Aβ40-Zn2+ oligomers. The images disagree with the quantitative data, especially when the compound is applied at an equimolar concentration.

The images are representative of the whole population, which contains 50-60 cells per condition. We have updated the legends of Figures 2 and 3 to clarify this point, with thanks to the reviewer for their attention to detail.

9. If the mechanism by which trodusquemine interfere with oligomer binding is by increasing the rigidity of the cell membrane, a correlation between rigidity and degree of inhibition would be expected. Can such a correlation be tested by examining a few other aminosterols? Would compounds that decrease membrane rigidity increase oligomer binding?

The reviewer brings up an interesting point that may be explored in future work, especially considering that relevant aminosterols are of limited availability from commercial sources and would need to be synthesized. Since our statement on the size and its relationship to bending rigidity is speculative and not essential for our main conclusions, we have removed it from the manuscript and revised its associated text (lines **251-252** of the revised version).

10. In Figure 4d, the increase in the static light scattering of HypF-N oligomers in the presence of different concentrations of trodusquemine appears to be substantial and consistent at all concentrations compared to HypF-N oligomers in the absence of trodusquemine. The conclusion that the compound has no effect does not appear to be supported by the data.

In the original version of the manuscript we observed that there was no appreciable difference between trodusquemine-exposed and unexposed HypF-N oligomers in Fig. 4d. To render this observation more quantitative, we have now added specific P values, which show there is indeed no significant difference between these populations, which is also in agreement with the turbidity data shown in Fig. 4c. We also note that markedly larger (and therefore statistically significant) changes in size are needed to attenuate oligomer cytotoxicity, as assessed by a well-quantified size-hydrophobicity relationship (Mannini et al., *ACS Chem. Biol*., 2014) and shown using several molecular chaperones (Mannini et al., *PNAS*, 2012; Mannini and Chiti, *Front. Mol. Neurosci.*, 2017). Our conclusion on the mechanism of action for trodusquemine is therefore consistent with the data. We have also reworded the sentence highlighted in Point 11 below to ensure our claims are consistent with the data.

11. The interpretation proposed in lines 245-246, "…the decrease in oligomer cytotoxicity is not mediated by a change in the oligomer structure…" is going too far. Later in the Discussion, the authors acknowledge that changes in oligomer structure may contribute to the

changes in toxicity, though disruption of the interaction with the membrane may be the main mechanism. This interpretation is in line with the data, but not the sentence cited above.

We have reworded the sentence accordingly in **lines 295-297** of the revised manuscript, with thanks to the reviewer.

12. DLS data are presented only for α-synuclein and AFM only for Aβ. These analyses should be done and presented for all three proteins.

We have characterized the size of all the protein misfolded oligomers in bulk using static light scattering and turbidity absorbance, whereby the two techniques are in complete agreement. We therefore carried out supplementary experiments using AFM on Aβ40 oligomers and DLS on α-synuclein oligomers to support these data, again observing that trodusquemine does not increase the size of the aggregates under these conditions, which if true could decrease their affinity for the membrane (reference Mannini et al., *ACS Chem. Biol.*, 2014 for the size-hydrophobicity relationship). We note that we have studied these oligomers extensively herein, having referenced at least four papers for each oligomer type, and validated their integrity regularly throughout our experiments. All data directly support with confidence our main conclusion that trodusquemine functions at these physiologically relevant concentrations to suppress toxicity predominantly via the displacement of oligomers from cell membranes.

13. The AFM analysis of Aβ shows clearly that the oligomer size distribution is shifted toward smaller ones in the presence of trodusquemine, in contrast to the claims in the text.

We have revised the corresponding sentence from saying that it is "not significantly changed" to "not significantly increased" in **line 291** of the revised text to match the title of Supplementary Figure 7 in the revised version. We note that a possible decrease in size mediated by trodusquemine cannot explain the decreased oligomer toxicity, because toxicity correlates negatively with oligomer size.

14. FTIR is not a very sensitive method for measuring small changes in secondary structure of amyloid proteins. Adding CD analysis will be more convincing. In addition, the protein concentration used in the FTIR and the large excess of trodusquemine make it impractical to correlate these data with other experiments. The same concentration ratios should be used as in all other experiments.

The reviewer is correct when the analysis involves the secondary structure of soluble proteins. However, FTIR is more sensitive than CD in the analysis of proteins in their aggregated states. We believe that the reason is because FTIR is more sensitive to detect β-sheets peaks that are well separated from any other secondary structure contribution, whereas in CD the β-sheet band is weak and often shadowed by α-helical bands. More importantly, CD suffers from differential absorption flattening, where the CD spectra are not just flattened, but also distorted if aggregates are present in the sample (Castiglioni et al., *Chirality*, 2007). Indeed, our sample is in the aggregated states since we utilized stabilized oligomers. This point has been addressed in **lines 306-309** of the revised manuscript.

We also note that we employed 1:10 excess ratios of the molecule because we saw minimal alterations in secondary structure for HypF-N and Aβ40 oligomers at lower concentrations. We have added **Supplementary Figure 8** to show one such experiment with only a 2.5-fold excess of trodusquemine with a corresponding line in the text (**lines 314-316** of the revised version). The previous and added data are consistent with our conclusions on the mechanism of action for trodusquemine in its ability to protect cells from oligomers.

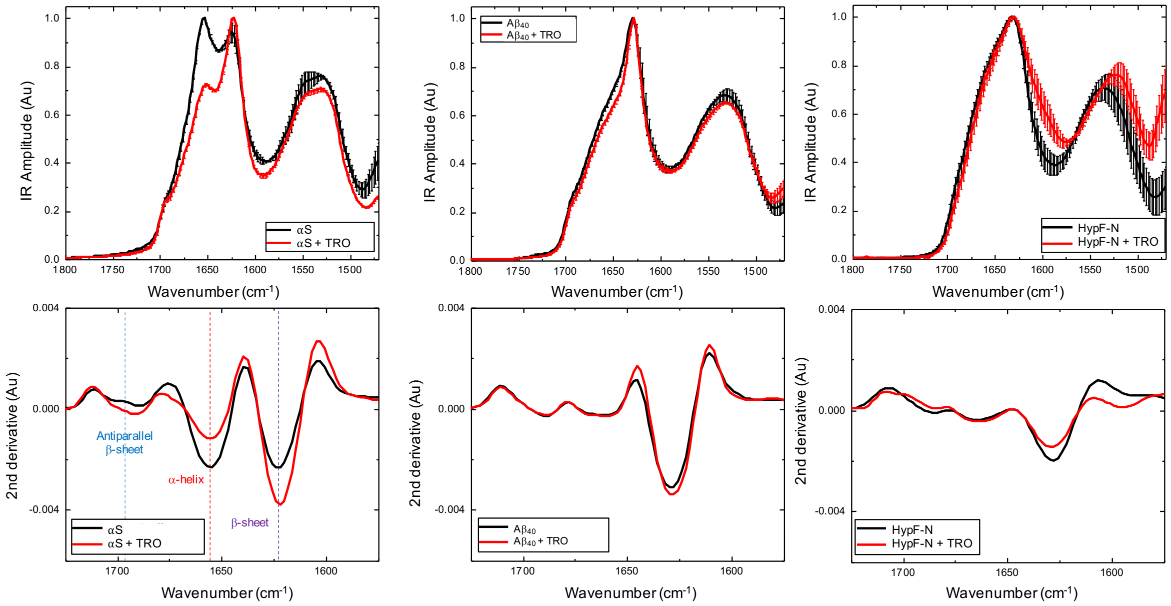

Supplementary Figure 8. Structural characterization of the various types of oligomers with a 2.5-fold excess of trodusquemine. *IR absorbance measurements of oligomers of αS (left panels), $A\beta_{40}$ (middle panels) and HypF-N (right panels) incubated in the absence (black traces) or presence of a 2.5-fold excess of trodusquemine (red traces). The spectra were acquired in triplicate and averaged (left panels) and the error bars indicate the s.e.m. of three replicates. All spectra were normalized to assign an arbitrary value of 1.0 Au to the maximum absorbance. Secondary derivative analysis of the averaged spectra (bottom panels), with key inflection points corresponding to antiparallel β-sheet (light blue), α-helix (red), and parallel β-sheet (purple) indicated with vertical dashed lines. The presence of a 2.5-fold excess of trodusquemine was observed to increase the β-sheet content and reduce the combined α-helix and random coil content of αS oligomers to a smaller extent in comparison to a 10-fold excess of trodusquemine (ref* ***Fig. 5****), while the overall structural compositions of $A\beta_{40}$ and HypF-N oligomers remained largely unchanged in the presence of the molecule as also observed at higher concentrations of the molecule.*

15. The hypothesis that charge contributes to the effect of trodusquemine, particularly “the negatively charged sialic acid of GM1” (lines 315-316) is interesting. Can it be tested experimentally?

This is an interesting point that may be explored systematically in future studies, as the scope of the current manuscript is to show evidence that several types of oligomers are displaced from the membrane by trodusquemine in a generic manner, resulting into a neutralization of

their toxicity without changing their overall structural and physicochemical properties.

Minor issues

1. WHO-mandated nomenclature is that Aβ is an abbreviation of amyloid β-protein, not amyloid-β peptide.

We have updated the manuscript accordingly, with thanks to the reviewer.

2. In line 219 the concentration of trodusqumine is described as "0.1, 0.33, and 1 molar equivalents" after in all previous instances it was described as "10:1, 3:1, and 1:1 ratios". For clarity, it would be better to use one consistent description.

We have revised the manuscript to take this point into account.

3. The concentration units used for the IR experiments, mg/mL, are inconsistent with all the other cases, where the concentration is given in μL. The authors should use consistent units throughout the manuscript.

We have updated the manuscript accordingly (**lines 523-524** of the revised manuscript), "Oligomers were prepared as above but at 2 mg/ml, a concentration of protein that is sufficient to resolve reproducible and sufficiently intense absorbance from the oligomers…"

4. References to Figures 5A and 5B are missing in the text.

We have updated the manuscript accordingly (**lines 311 and 319** of the revised manuscript).

5. In the sentence starting with "After washing with PBS…" in line 398, the mention of HypF-N oligomers is missing.

We have updated the manuscript accordingly (**line 467** of the revised manuscript).

6. The grammar of the first sentence in the ANS binding measurements (line 414) should be corrected.

We have updated the manuscript accordingly (**lines 483** of the revised manuscript).

7. The "Light scattering" section (starting at line 432) is confusing. It starts with SLS and continues with DLS. Separate sections should be included for the two techniques.

We have updated the manuscript accordingly (**lines 499-508** of the revised manuscript).

REVIEWERS' COMMENTS:

Reviewer #1 (Remarks to the Author):

The reviewers have addressed all my comments and concerns and the paper has improved as a result of that. I am happy to recommend the article for publication.

Reviewer #2 (Remarks to the Author):

I appreciate the authors' responsiveness to the comments. They have addressed all the comments appropriately.
